# 3D virtual histology of rodent and primate cochleae with multi-scale phase-contrast X-ray tomography

**DOI:** 10.1038/s41598-025-89431-0

**Published:** 2025-03-07

**Authors:** Jannis J. Schaeper, Christoph A. Kampshoff, Bettina J. Wolf, Lennart Roos, Susann Michanski, Torben Ruhwedel, Marina Eckermann, Alexander Meyer, Marcus Jeschke, Carolin Wichmann, Tobias Moser, Tim Salditt

**Affiliations:** 1 Institute for X-Ray Physics, University of Göttingen, 37077 Göttingen, Germany; 2 Department of Otolaryngology, University Medical Center Göttingen, 37075 Göttingen, Germany; 3 Institute for Auditory Neuroscience and Inner Ear Lab, University Medical Center Göttingen, 37075 Göttingen, Germany; 4 Max Planck Institute for Multidisciplinary Sciences, 37075 Göttingen, Germany; 5 Molecular Architecture of Synapses Group, Institute for Auditory Neuroscience and InnerEarLab, University Medical Center Göttingen, Göttingen, Germany; 6 Beamline ID16A, European Synchrotron Radiation Facility, 38000 Grenoble, France; 7 Cognitive Hearing in Primates Group, German Primate Center, 37077 Göttingen, Germany; 8 Auditory Neuroscience and Synaptic Nanophysiology Group, Max-Planck-Institute for Multidisciplinary Sciences, Göttingen, Germany; 9 University Medical Center Göttingen, Center for Biostructural Imaging of Neurodegeneration, Göttingen, Germany; 10 Else-Kröner-Fresenius Center for Optogenetic Therapies, University Medical Center Göttingen, 37075 Göttingen, Germany; 11 Cluster of Excellence “Multiscale Bioimaging: From Molecular Machines to Networks of Excitable Cells”, University of Göttingen, 37075 Göttingen, Germany

**Keywords:** Cochlea imaging, X-ray tomography, Synchrotron radiation, Micro-CT, Phase contrast, Spiral ganglion neurons, Biophysics, Imaging techniques, X-rays

## Abstract

Multi-scale X-ray phase contrast tomography (XPCT) enables three-dimensional (3D), non-destructive imaging of intact small animal cochlea and apical cochlear turns. Here we report on post-mortem imaging of excised non-human primate and rodent cochleae at different ${\upmu }$-CT and nano-CT synchrotron instruments. We explore different sample embeddings, stainings and imaging regimes. Under optimized conditions of sample preparation, instrumentation, imaging protocol, and phase retrieval, high image quality and detail level can be achieved in 3D reconstructions. The showcased instrumentation and imaging protocols along with the reconstucted volumes can serve as benchmarks and reference for multi-scale microanatomy and 3D histology. The provided benchmarks and imaging protocols of this work cover a wide range of scales and are intended as augmented imaging tools for auditory research.

## Introduction

The organ of Corti (OoC) in the mammalian cochlea is the receptor organ for hearing, which transduces sound to auditory nerve signals based on mechanoelectrical transduction by the hair cells (HCs) and synaptic transmission to spiral ganglion neurons (SNGs). In view of the intricate micro-anatomy and cyto-architecture of the OoC and the entire cochlea, non-destructive three-dimensional (3D) imaging techniques are required, both for studying fundamental aspects of signal transduction and neurophysiology as well as for preclinical research on hearing disorders. 3D imaging can also support the development of novel treatments, including for example auditory prostheses such as cochlear implants (CIs). In 3D imaging of these systems it is particularly relevant to bridge the micro-anatomy and histology, specifically of the OoC, and more generally the cochlea or even the entire temporal bone while non-destructively preserving its integrity. 3D imaging should hence be implemented on multiple length scales: while it is advantageous to cover the entire temporal bone to identify basic morphology or CI positioning for example, the isolated intact cochlea is studied for tonotopic mapping. Tissue samples of an apical cochlear turn, on the other hand, are required for studies of HCs and their innervation.

It is, however, noteworthy to address the critical aspects which deserve attention regarding the practical and technical limitations of multi-scale imaging. Achieving progressively higher resolution from micro- to nano-tomography comes at a price. It inherently restricts the sample size that can be imaged, necessitating meticulous sample preparation, including dissection and other preparatory procedures to isolate relevant regions of interest. Importantly, before such a study one must consider the invasive nature of such preparations and plan accordingly to ensure that the multi-scale imaging workflow is optimized and that critical structures are not inadvertently compromised. Furthermore, achieving the extreme precision necessary for nanotomography also demands specialized sample stages with highly accurate but limited movement capabilities. Finally, higher resolution extends scanning time, which should be considered when planning multi-scale imaging.

X-ray phase contrast tomography (XPCT) as a non-destructive and multi-scale 3D imaging technique can meet these needs^1^. It is compatible with a wide range of tissue preparation protocols, such as liquid embedding of unstained tissue^2^, paraffin-embedded tissue^3^, heavy-metal stained tissue^4,5^, decalcified and/or optically cleared samples^6,7^. However, corresponding to each case, instrumental settings, in particular photon energy, as well as phase retrieval schemes have to be well chosen and adapted to the preparation type and tissue size. 3rd and 4th generation synchrotron radiation (SR) facilities generate X-ray beams with a high degree of spatial and temporal coherence. This coherence is a prerequisite for exploiting the sample-induced phase shifts which are brought about by the decrement $\delta (\textbf{r})$ of the X-ray refractive index $n(\textbf{r}) = \delta (\textbf{r})-i\beta (\textbf{r})$. $\delta (\textbf{r})$, where $\textbf{r}$ denotes the position vector, is orders of magnitude higher than $\beta (\textbf{r})$ which is related to absorption, thus enhancing contrast for soft tissue. Phase shifts are converted by free-space propagation over a distance $z_{12}$ into measurable intensity distributions which can then be recorded by different types of detectors, which again have to be selected in view of the type of sample and the optical setup. For divergent X-ray beams, geometric magnification *M* is exploited in the setup, the physical detector pixelsize $\textrm{dx}$ thus translates into an effective pixelsize or voxelsize $\textrm{dx}_\textrm{eff} = \textrm{dx}/M$. The imaging regime is described by the unitless Fresnel number $F = \textrm{dx}_\textrm{eff}^2 / (z_\textrm{eff}\lambda )$ with the effective propagation distance $z_\textrm{eff} = z_{12}/M$ and photon wavelength $\lambda$^1^. At beamlines with undulator insertion devices, dedicated optics and endstations, such as the nanoimaging beamline ID16A/ESRF^8^ and the coherence beamline P10/DESY^9^, high *M* can be implemented, resulting in a phase imaging at $F\ll 1$, the so-called holographic regime. This in turn offers the advantage of high contrast even for weakly contrasted and small soft tissue components. Wiggler insertion devices such as at the biomedical beamline ID17/ESRF^10^, on the other hand, use a white beam with a broad spectrum with particularly high photon flux. Such instruments can be desirable for high-throughput recordings of larger samples, typically in the so-called direct contrast regime at $F\approx 1$. Finally, translation of phase contrast imaging to compact ${\upmu }$-CT instruments with low coherence is desirable for preclinical research.

A number of XPCT studies have already been devoted to post mortem imaging of the cochlea in different animal models. Here, the mouse cochlea was recorded to verify CI positioning within the cochlea by ${\upmu }$-CT^11^ and to study the cochlear morphology both with SR^12^ and ${\upmu }$-CT^13^. The guinea pig cochlea was recorded to visualize noise-induced hearing loss, exploiting the contrast of the osmium tetroxide stain for nervous tissue^14,15^. Further, the cochlear morphology of the guinea pig and marmoset cochlea was imaged with a compact synchrotron source^16^. The distinct cochlear morphology was then compared between several animal models with ${\upmu }$-CT^7^. Using SR for the marmoset model, particularly high image quality was reached at $0.65\,{\upmu }\hbox {m}$ voxel size^7^. Recently, XPCT was also applied to the human cochlea, using both SR^17–19^ and ${\upmu }$-CT sources^13,15^, for which the tissue was stained with osmium tetroxide. Advanced image segmentation tools have been used to analyze and exploit these results^17^.

**Fig. 1:**
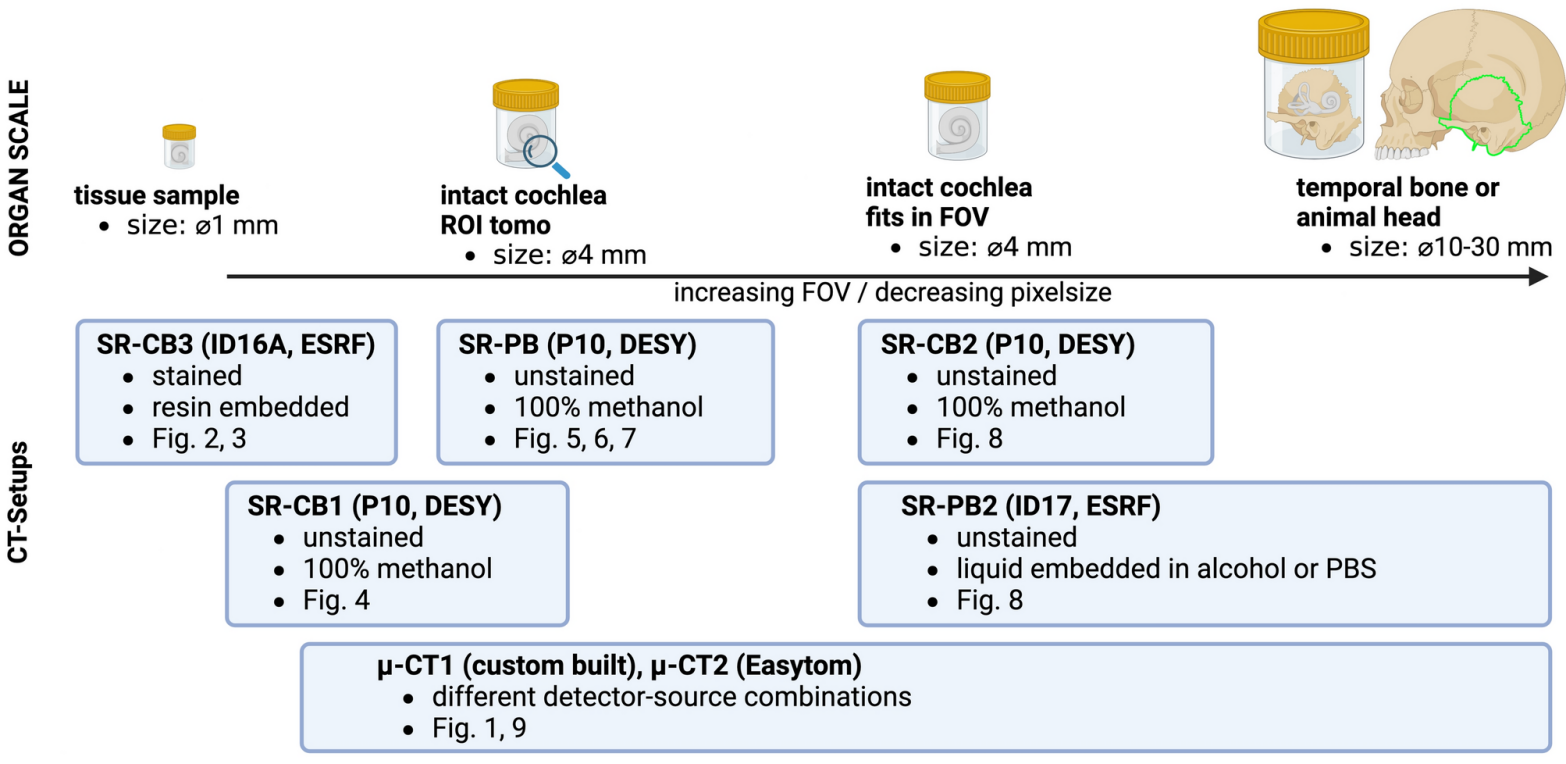
Orientation panel for the different applications and the corresponding instrumental settings, presented in this work. *Created in BioRender. S, J. (2025) https://BioRender.com/b49j773*.

In this work, we evaluate the use of XPCT for imaging non-human primate and rodent cochlea. The goal of this work is primarily to explore different approaches of sample preparation and instrumental settings, including both SR and inhouse computed tomography with microfocus X-ray sources (${\upmu }$-CT). To this end, we cover a wide range of setups, preparation protocols and phase retrieval approaches. Instead of comparing everything to everything, we select different prototypical configuration, for which we found conditions which ‘matched’ best. Since the different instruments and configurations can easily become somewhat confusing for the reader, we have included an orientation panel with reference to the figures further below, see Fig. 1. With this work, it is also our intention to generate and deposit reference datasets of different cochleae and imaging configurations, which may be helpful for researchers in the field to design their own experimental study, or for those which need a 3D reconstruction for modeling.

Following this introduction, the results section below addresses the different sample classes and multi-scale imaging approaches, starting from smaller to larger specimen: First, we present results for the organ of Corti, scanned at high resolution setups and voxelsizes in the range of 50 nm to 170 nm at ID16A/ESRF and P10/DESY, resolving single cells such as the inner hair cells (IHCs). Suitable instrumental settings are given for imaging of unstained tissue as well as for stained tissue. In the latter case, high contrast is achieved for nervous tissue, down to small fibers. Next, region-of-interest (ROI) tomography of the intact cochlea is presented for marmoset and mouse, reaching a resolution level high enough for segmentation e. g. of the spiral ganglion neurons (SGNs). Finally, results are presented for the entire cochlea, scanned at ${\upmu }$m resolution to study basic cochlear morphology and CI positioning. The MS closes with a discussion, brief summary and outlook, followed by the detailed methods section.

## Results

### High resolution virtual histology of the organ of Corti

**Fig. 2:**
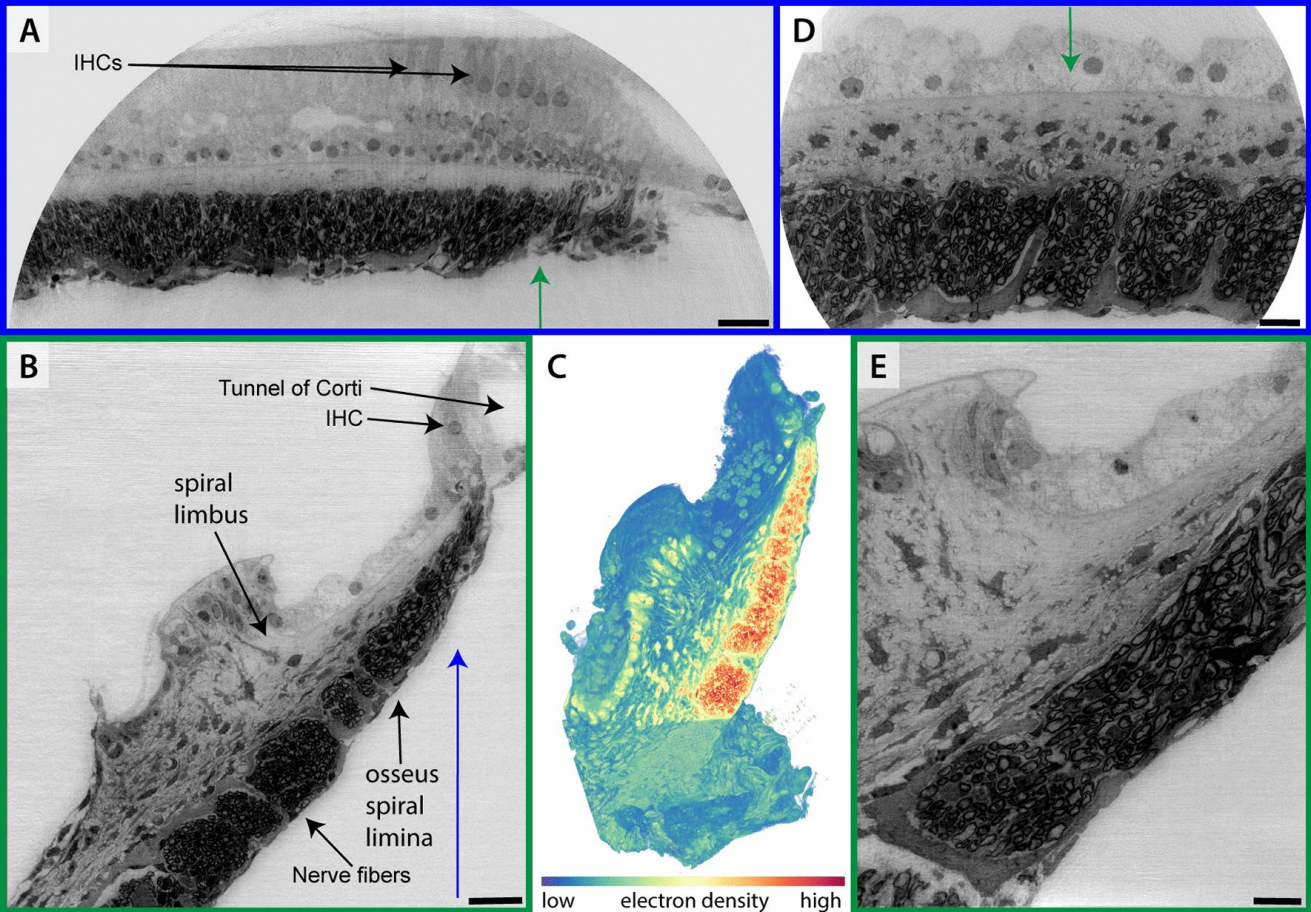
High resolution recording of osmium uranylacetate stained and EPON embedded apical cochlear turn of a mouse with part of the OoC, spiral limbus and osseus spiral lamina containing nerve fibers of SGNs projecting to HCs (sample Mo1) at *SR-CB3*. (A) Overview scan, the row of IHCs is clearly visible. (B) Virtual reconstructed slice perpendicular to (A) at the position of the green arrow, showing an IHC and the tunnel of Corti. (C) 3D Rendering of the dataset presented in (A,B) (NVIDIA IndeX). (D) ROI-tomography at smaller voxelsize, same orientation as (A). (E) Virtual reconstructed slice perpendicular to (D) as indicated by blue arrow, with higher resolution than (B). (A-C) $\textrm{dx}_\textrm{eff} = 115\,\hbox {nm}$, scalebars $25\,{\upmu }\hbox {m}$, (D,E) $\textrm{dx}_\textrm{eff} = 50\,\hbox {nm}$, scalebars $10\,{\upmu }\hbox {m}$. As indicated by the colors of the panel frame, (A,D) show the same orientation, and (D,C,E) are orthogonal. At the same time, the zoom levels are also grouped into (left hand side) $\textrm{dx}_\textrm{eff} = 115\,\hbox {nm}$ (A,B,C) and (right hand side) $\textrm{dx}_\textrm{eff} = 115\,\hbox {nm}$ (D,E)..

**Fig. 3:**
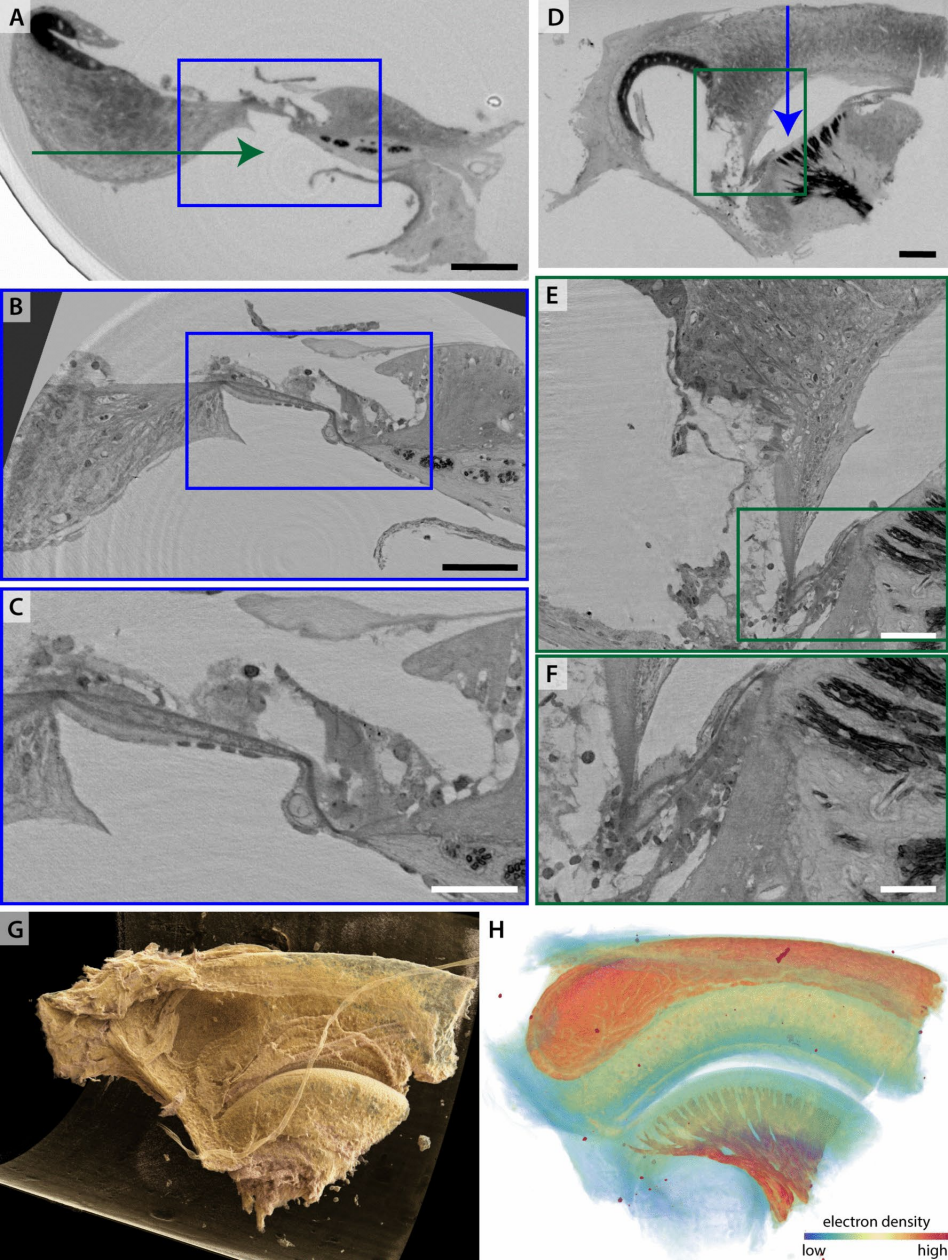
High resolution XPCT of osmium stained apical cochlear turn (sample Mo2). (A,D) ${\upmu }$-CT2 overview scan at $\textrm{dx}_\textrm{eff} = 1\,{\upmu }\hbox {m}$, the orientation of the perpendicular slices is indicated with arrows. (B,E) High resolution *SR-CB3* scan at $\textrm{dx}_\textrm{eff} = 115\,\hbox {nm}$. (C,F) Zoom into (B,E) respectively. (G,H) Volume renderings of the unsegmented overview scan presented in (A,D). The renderings shown in (G,H) cover a lateral FOV of 0.6$\times$0.8 mm$^2$. Scale bars top row $100\,{\upmu }\hbox {m}$, middle row $50\,{\upmu }\hbox {m}$, bottom row $25\,{\upmu }\hbox {m}$.

First, we present imaging results for OoC specimen which have been carefully dissected from mouse and marmoset cochleae. Both stained and resin embedded as well as unstained and liquid embedded OoC samples were investigated.

Fig. 2 shows results of a mouse tissue sample (Mo1) which was stained with osmium tetroxide and uranylacetate staining, and embedded in EPON (epoxy resin), following conventional protocols used for TEM. In the present case, however, EPON was cast into a cylindrical shape in order to reduce artefacts from edges and to have similar absorption for all projections. The specimen was scanned in the *SR-CB3* configuration, and was selected and prepared in order to be compatible with the vacuum environment and high photon energy $E = 17.1\,\hbox {keV}$ available at this instrument. Since the EPON embedding is relatively stable, this also allowed for long scan times and scanning at several defocus distances, as required by optimal phase retrieval based on the contrast-transfer function (CTF) approach. A description of the setups and a list of all acquisition parameters can be found in the methods section. Scans were acquired at different voxelsizes $\textrm{dx}_\textrm{eff} \in \{50\,\hbox {nm}, 80\,\hbox {nm}, 115\,\hbox {nm}\}$. Virtual slices through the reconstruction volume show a high level of detail, clearly with subcellular resolution. The row of IHCs as well as the spiral limbus is well resolved. The osmium uranylacetate stain enhances contrast especially for the lipid-rich nerve fibers that can be traced through the entire volume of the osseus spiral lamina. The intensity-based 3D volume rendering shows the extension of the OoC through a part of the corresponding cochlear turn. The sample was well-preserved and did not move during the measurement.

Next, a second osmium stained and EPON embedded mouse apical turn (sample Mo2) is shown in Fig. 3. Here, about a third of a turn was dissected. The sample was first scanned inhouse at ${\upmu }$-CT2, see slices shown in (A,D), corresponding renderings are depicted in (G,H), and subsequently the sample was also scanned at *SR-CB3* for high resolution (B,C,E,F). The photo-realistic rendering in (G) gives an impression of how the sample was cut. The in-house ${\upmu }$-CT scan recorded at a tube voltage $U = 80\,\hbox {kV}$ and pixel size $\textrm{dx}_\textrm{eff} = 0.44\,{\upmu }\hbox {m}$ already yields a surprisingly high image quality sufficient to control the quality of the sample preparation protocol and to select positions for subsequent SR-ROI tomography. The *SR-CB3* scan acquired at $\textrm{dx}_\textrm{eff} = 125\,\hbox {nm}$ and a photon energy $E = 17.1\,\hbox {keV}$ was manually aligned to the overview scan. Cellular resolution is reached with the darker areas showing the stained nervous tissue. This high image contrast for the stained nerve tissue facilitates visualization in 3D within a ray tracing rendering framework (NVIDIA IndeX), see 3(H).

**Fig. 4:**
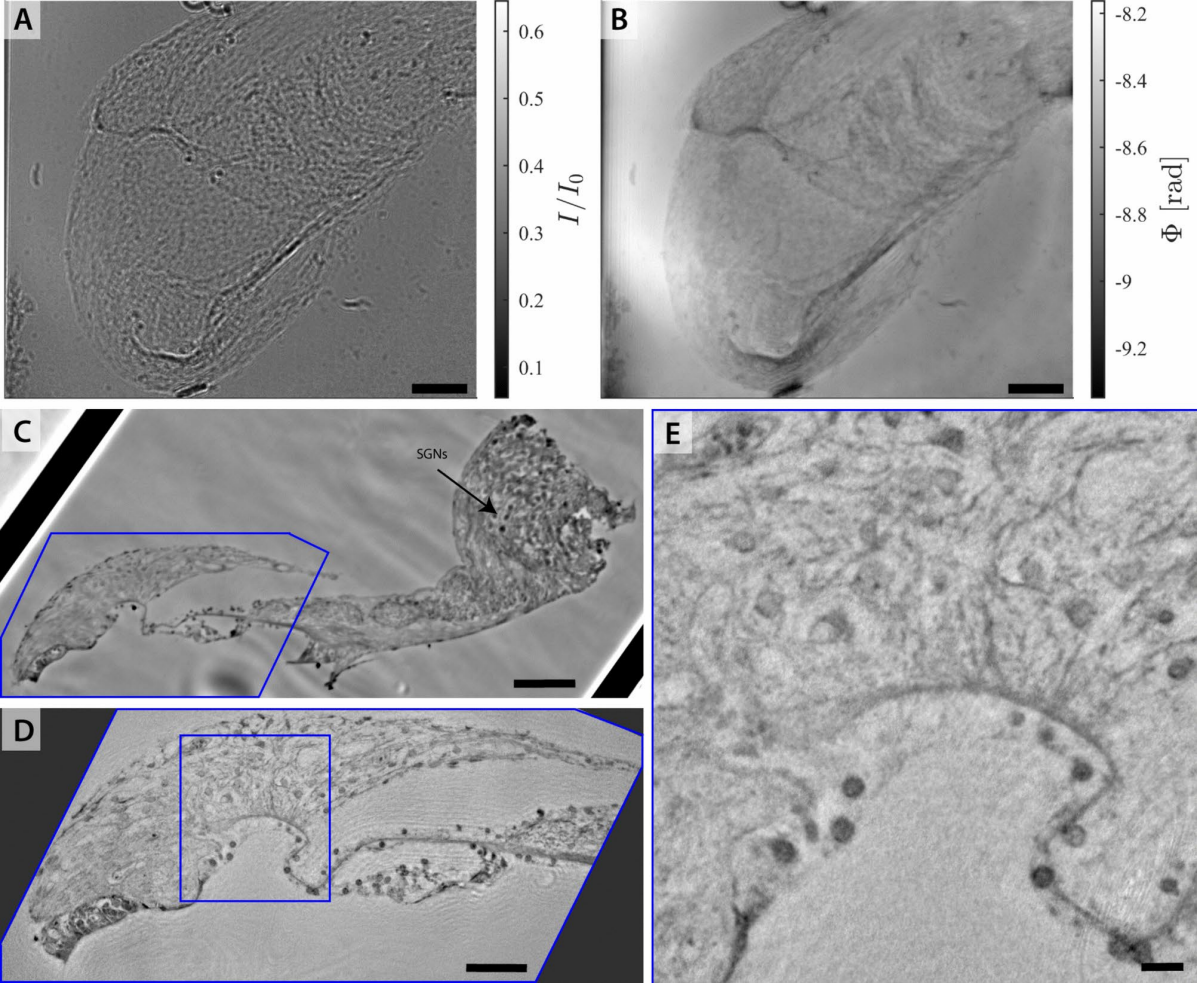
XPCT of marmoset’s OoC tissue in 100 % methanol (sample Ma5). (A) Typical projection image acquired in the *SR-CB1* configuration. (B) Phase retrieval of (A) with CTF-scheme. (C) Virtual slice through the reconstruction volume of an overview scan acquired with the *SR-PB* configuration, scalebar $100\,{\upmu }\hbox {m}$. (D) ROI-tomography of (C) with *SR-CB1* setup, position with respect to (C) as indicated by the blue rectangle, scalebar $50\,{\upmu }\hbox {m}$. (E) Zoom into (D) as indicated by blue rectangle, scalebar $10\,{\upmu }\hbox {m}$.

The third OoC tissue sample presented in this section is unstained and stems from a decalcified marmoset cochlea (sample Ma5). The results are shown in Fig. 4. Here, a part of the cochlear turn was dissected, transferred to a glass tube of a diameter of 1 mm, and immersed in 100 % methanol. This preparation is incompatible with measuring in vacuum, but since the tissue is unstained, it is well-contrasted at lower photon energies. As this embedding is not as stable, sample movement and evaporation of the solvent can occur. Thus, fast acquisition is desirable and only a single defocus distance was recorded. The sample was first scanned in a parallel beam (PB) setup *SR-PB* at $E = 13.8\,\hbox {keV}$, see panels (A-C). The high flux density in this configuration enables the recording of a tomogram at sub-${\upmu }$m voxelsize in 1.2 min. The overview scan shows the whole sample already at a high detail level. The SGNs are well resolved. Target regions for a subsequent ROI-tomogram at smaller voxelsize can be conveniently selected. Such ROI-scans were then recorded in a cone-beam geometry (*SR-CB1*), see panels (D,E), at $\textrm{dx}_\textrm{eff} = 167\,\hbox {nm}$ and $E = 8\,\hbox {keV}$. The ROI-dataset was manually aligned to the overview scan for orientation. Slices through the reconstruction volume show the OoC and the innervations with the SGN. However, it also becomes apparent that the tissue sample got distorted by the preparation, pointing at the challenge of OoC dissection and preparation.

### ROI-tomography of the intact marmoset and gerbil cochlea at sub-${\upmu }$m resolution

**Fig. 5:**
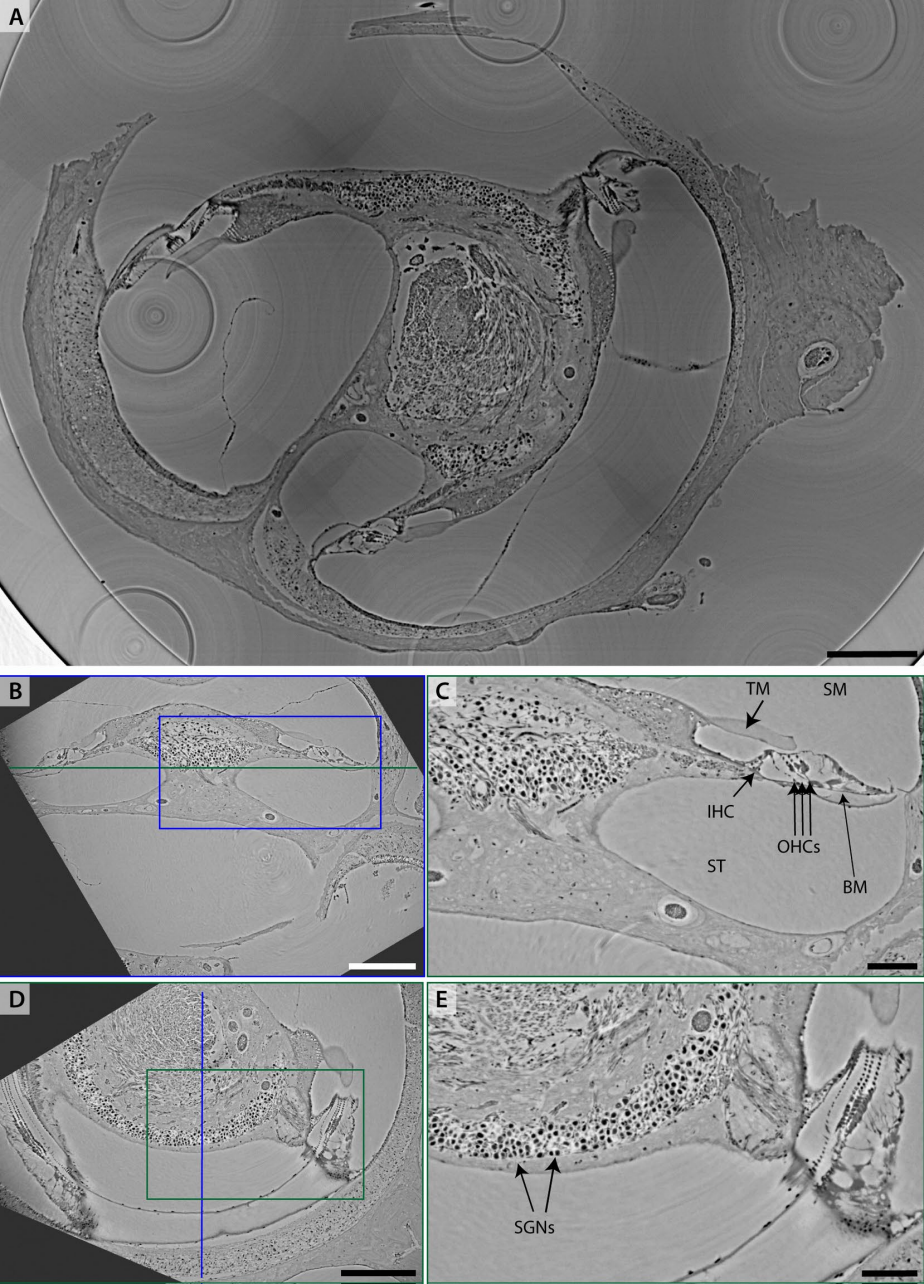
Imaging the intact gerbil cochlea (Ge1) by recording $3\times 3\times 4$ ($x\times y\times z$) ROI-tomograms and subsequent stitching. (A) Virtual slice through the reconstruction volume after stitching 9 tomograms in the second plane. This volume is binned 4-fold and acts as a low resolution reference for the single tomograms that are recorded with sub-${\upmu }$m resolution. (B,D) Perpendicular slices through the reconstruction volume of the central tomogram. Note that this volume has been rotated so that the modiolus is upright. (C) Zoom into (B) and (E) zoom into (D) to showcase the image quality. The SGNs are highly contrasted to the surrounding tissue. The IHCs and OHCs in the OoC are visible. The cochlear morphology with the scalae (scala tympani (ST), scala vestibuli (SV), scala media (SM)) and separating membranes (Reissner’s membrane (RM), basilar membrane (BM) and tectorial membrane (TM)) is represented well. The datasets have been recorded at $\textrm{dx}_\textrm{eff} = 650\,\hbox {nm}$. Scalebars (A,B,D) 0.3 mm, (C,E) 0.1 mm.

Next we explore the fast acquisition scheme at the *SR-PB* setup to image the intact rodent and non-human primate cochlea, by stitching tomograms. A gerbil cochlea (Ge1) was fixated, decalcified and parts of temporal bone was removed. The sample was mounted with agarose in an Eppendorf cup and immersed in 100 % methanol. Trapped air bubbles were removed by applying a low vacuum in a vacuum oven. The whole sample was covered with ROI-tomograms. Before recording each plane in a 3-by-3 mesh, flatfield images were recorded to account for changes in illumination. The whole sample was covered by recording the cochlea in 4 planes, resulting a total of 36 tomograms. The projections were flatfield corrected with the closest set of flatfield images. A CTF-based single-distance phase retrieval was performed. Each tomogram was reconstructed separately by filtered backprojection. Fig. 5(A) shows a slice of the second 3-by-3 plane, stitched with the *NRStitcher*^20^ and binned 4-fold. This does give an overview of the sample and can act as a low-resolution lookup-table. Data analysis is then performed on the single tomograms which are referenced by the stitched volume. In panels Fig. 5(B-E), perpendicular virtual slices through the reconstructed volume of the central tomogram are shown. This volume has been rotated to match the orientation of typical schematic representations of the cochlea with the modiolus being positioned upright in the middle. The dataset shows cellular resolution. The SGNs and the HCs in the OoC are particularly well contrasted. All membranes and scalae are also well represented. The liquid embedding worked well for the sample here, no motion artefacts or bubble formation is visible which would otherwise introduce severe artefacts.

**Fig. 6:**
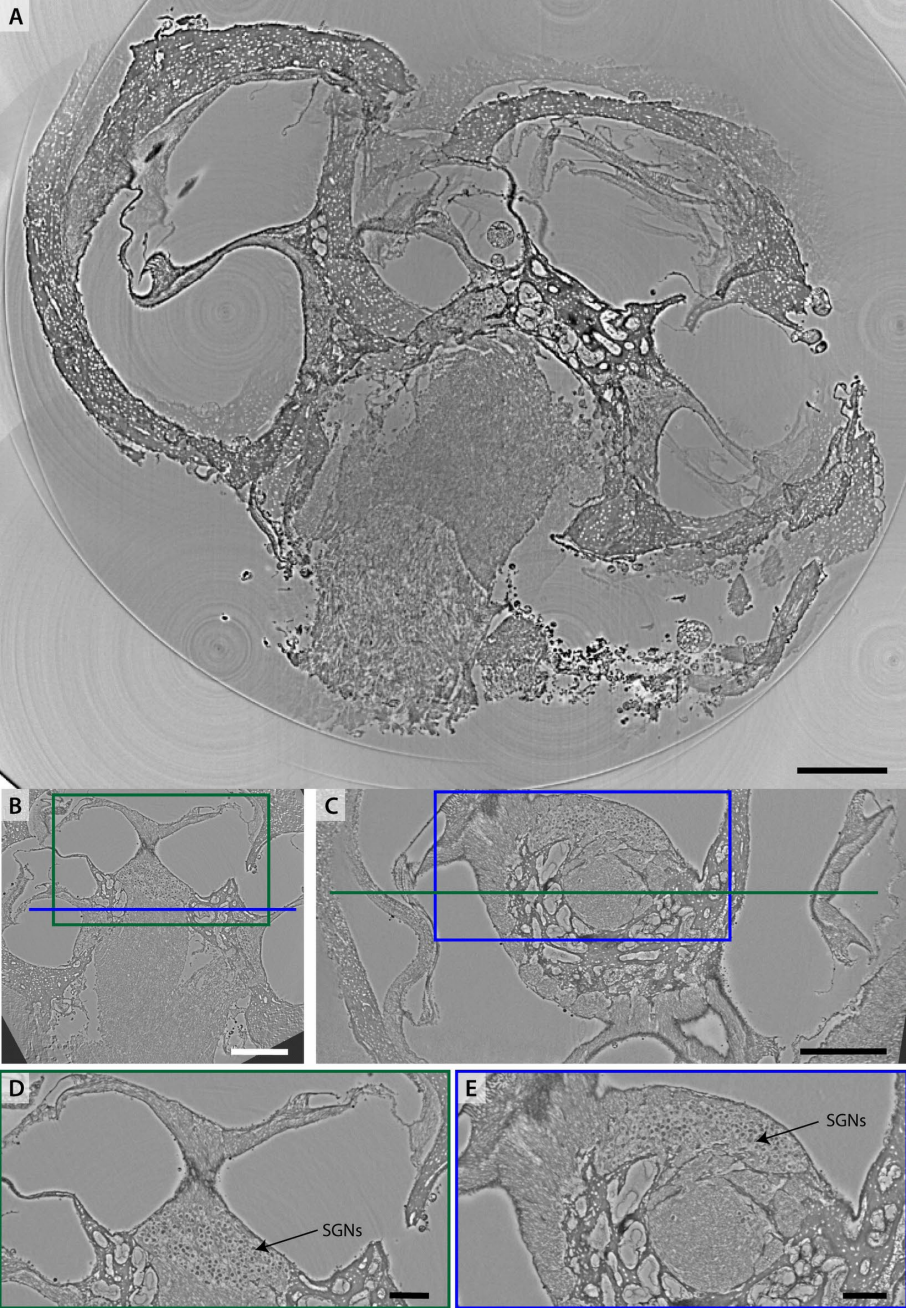
Imaging the intact optically cleared marmoset cochlea (sample Ma3) by $3\times 3\times 4$ ($x\times y\times z$) ROI-tomograms and subsequent stitching. (A) Virtual slice through reconstruction volume after stitching 9 tomograms in a plane. Note that while this stitching is imperfect it gives an impression of the whole sample plane. This volume is binned 4-fold. (B,C) Perpendicular slices through the central volume of the plane, rotated to the axis/planes of principal symmetry of the cochlea. (D) Zoom into (B) and (E) zoom into (C). The SGNs separate through a high contrast from the surrounding tissue. Scalebars (A-C) 0.3 mm, (D,E) 0.1 mm.

**Fig. 7:**
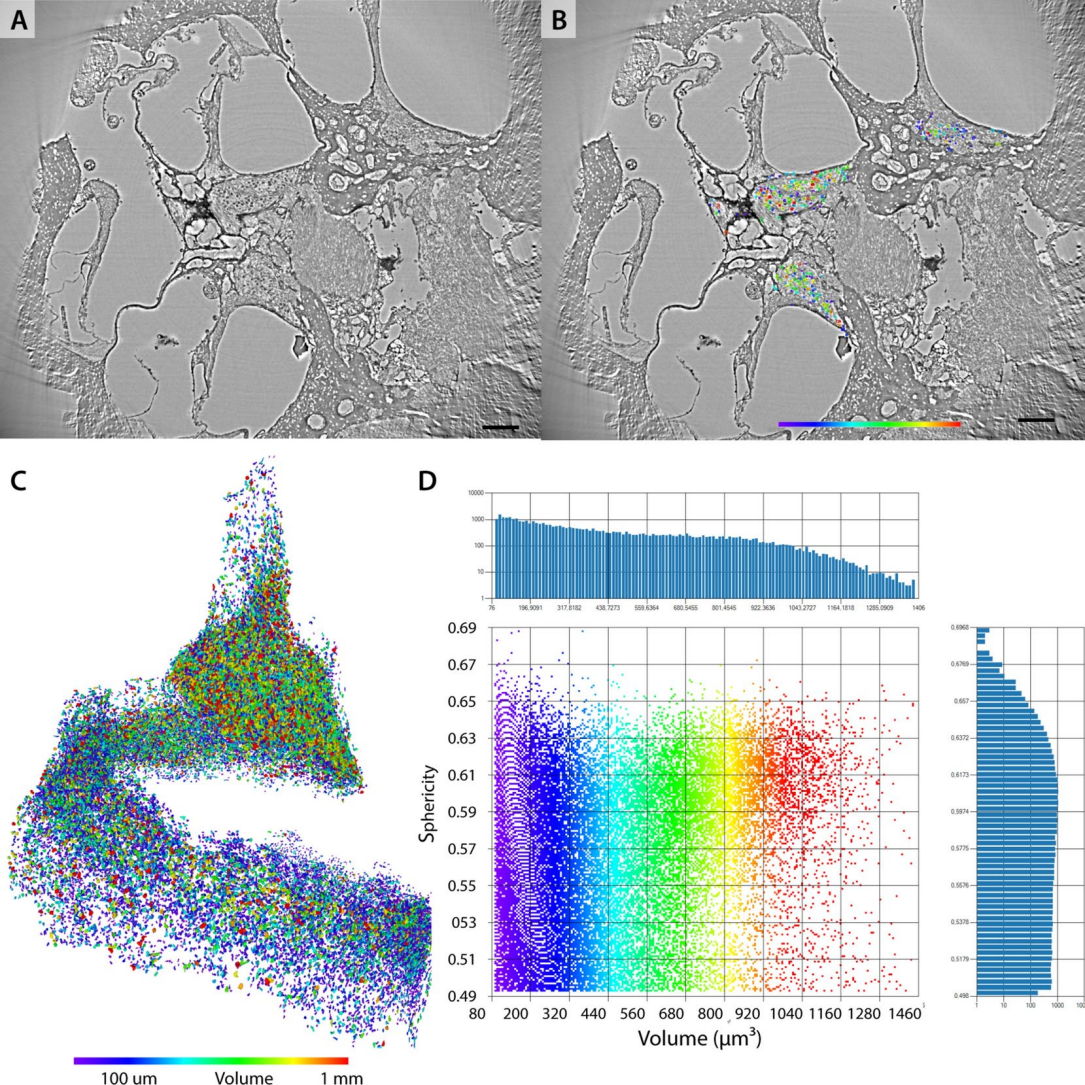
Segmentation of SGNs in a subvolume of the scan introduced in Fig. 6 acquired at *SR-PB* of the marmoset cochlea (sample Ma3). (A) Virtual slice through the reconstruction volume of the central tomogram. (B) Same slice as (A), the segmented SGNs are coloured. (C) 3D rendering of all segmented SGNs. (D) Scatterplot of the features sphericity and volume. Scalebar (A,B) 0.2 mm. Colourmap volume $0.1\,\hbox {mm}^{3}$ to $1\,\hbox {mm}^{3}$.

Further, we scanned an intact marmoset cochlea (Ma3) with the same protocol. The cochlea was fixated, decalcified, optically cleared for light sheet fluorescence microscopy (LSFM) and immersion embedded in dibutyl ether (DBE). With the *SR-PB*-setup, the whole cochlea was covered with 36 ROI-tomograms at $\textrm{dx}_\textrm{eff} = 650\,\hbox {nm}$ and $E = 13.8\,\hbox {keV}$. Fig. 6 shows virtual slices through the stitched reconstruction volume and the central ROI-tomogram of the second plane. The overview here again serves as a look-up table for the correct subvolumes. Note that since the stitching did not work perfectly, the outer circle does not close. The single tomograms can be recognized by the slight ring artefacts – the center of each ring cascade is a tomogram. In Fig. 6 (B) and (C), perpendicular slices through the central tomogram of the plane are shown. Again, the volume was rotated to match typical representations of the cochlea with the modiolus facing upwards. The cochlear morphology is represented well with the scalae, membranes and the OoC. Further, the SGNs show a high contrast facilitating their segmentation.

In view of segmentation, the SGN nuclei can be treated as dense and compact (roughly spherical) objects in the 3D volume, which can for example be identified by the so-called blob-finder algorithm (Arivis, ZEISS Microscopy, Oberkochen, Germany). After automated seed finding, a watershed transformation was performed to separate the objects. Then, a 3D mask was drawn manually in some slices, to mark the area in which the SGNs are located. For the rest of the slices, the mask was interpolated. Accordingly, all objects are enclosed within the masked region. Subsequently, by visual inspection, the object parameters *sphericity* and *volume* were restricted to typical SGNs morphology, ruling out most of the false-positively identified SGN, classified as non-SGN-blob. Fig. 7 shows an example slice of the central volume with and without the segmented SGNs. Further, a 3D representation of the segmented SGNs is shown. Note that the shape and density distribution of the neuronal nuclei can represent interesting histological parameters, as shown before for the central nervous system^21,22^. However, finite pixel size and noise, as well as errors in segmentation can act as a confounder. For this reason, the histogram has to be carefully inspected and tighter thresholds may have to be used. Also the segmentation process has to be carefully evaluated.

### Overview scans of intact marmoset cochleae

**Fig. 8:**
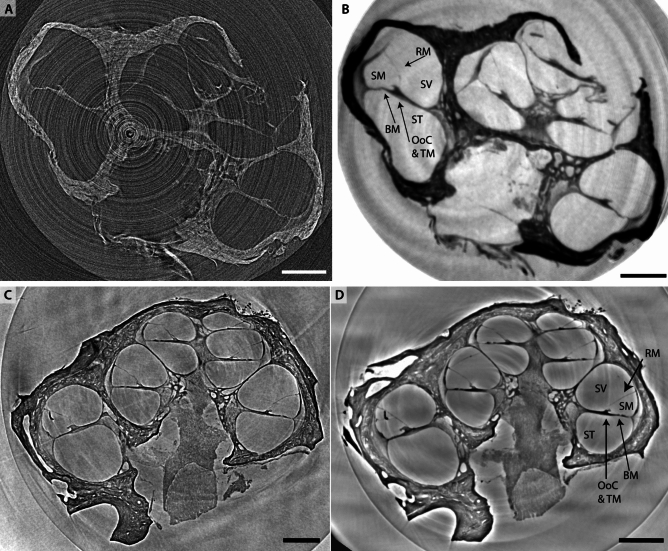
XPCT overview scans of the cleared marmoset cochlea (top sample Ma3, bottom sample Ma4). (A,B) Demonstration of phase contrast for in-house ${\upmu }$*-CT1* at $\textrm{dx}_\textrm{eff} = 4.15\,{\upmu }\hbox {m}$ where (A) tomographic reconstruction without prior phase retrieval and (B) tomographic reconstruction after phase retrieval with BAC-scheme. (C) Acquired at *SR-CB2* at $\textrm{dx}_\textrm{eff} = 3.05\,{\upmu }\hbox {m}$. (D) Acquired at *SR-PB2* at $\textrm{dx}_\textrm{eff} = 3.58\,{\upmu }\hbox {m}$. In all three slices the basic cochlear morphology such as the three scalae (scala tympany (ST), scala media (SM) and scala vestibuli (SV)) and the separating membranes (Reissner’s membrane and basilar membrane with the OoC and tectorial membrane (TM)) are clearly represented. Note that in (a) grey values are attenuation-weighted, with white representing higher absorption. Contrarily, in (b) the contrast is electron density weighted, and as all images involving treatment of phase effects is is shown with black representing highest density. Due to the lack of quantitative values/calibration, we refrain, however, from indicating colorbar values. Scalebars $500\,{\upmu }\hbox {m}$.

Next, we illustrate the importance of phase-contrast and phase retrieval for in-house ${\upmu }$-CT of the entire chochlea, and place this into context with synchrotron scans, equally at the scale of large overview scans. To this end, we selected a cleared marmoset cochlea (Ma3) mounted in an Eppendorf cup with agarose and immersed in DBE. The $\mu$*-CT1* instrument was operated at a tube voltage of 70 kV. The setup was equipped with a flat panel detector placed at a propagation distance $z_{12} = 2\,\hbox {m}$, resulting in a geometric magnification $M = 18$ and a voxelsize $\textrm{dx}_\textrm{eff} = 4.15\,{\upmu }\hbox {m}$. At $F = 1.37$, we are in the direct contrast regime where phase contrast manifests itself as edge enhancement, see typical projections in the Supplemental. Phase retrieval, notably with the BAC-scheme^23^ considerably improves image quality of the tomographic reconstruction, see the comparison in Fig. 8(A,B). When prior phase retrieval is performed, the tomographic reconstruction of this cleared cochlea exhibits high contrast and detail level. The basilar membrane is well represented, and in most of the slices also Reissner’s membrane is visible. As a comparison, virtual slices through the reconstruction volume of the cleared marmoset cochlea (Ma4) acquired at *SR-CB2* and *SR-PB2* are also shown in (C) and (D), respectively. The *SR-CB2* dataset in (C) was recorded at a monochromatic energy at $E = 13.8\,\hbox {keV}$ with the sample 34 mm in front of the detector, corresponding to $F = 0.32$. Phase retrieval was performed with the CTF- approach^24^. Contrarily, the *SR-PB2* in (D) uses a wiggler as photon source radiating SR with a broad spectrum and peak energy $E = 50\,\hbox {keV}$. For the peak energy, the Fresnel number is $F = 0.52$. Phase retrieval was performed by the generalized Paganin method (GPM)^25^.

### Marmoset head with eCI

**Fig. 9:**
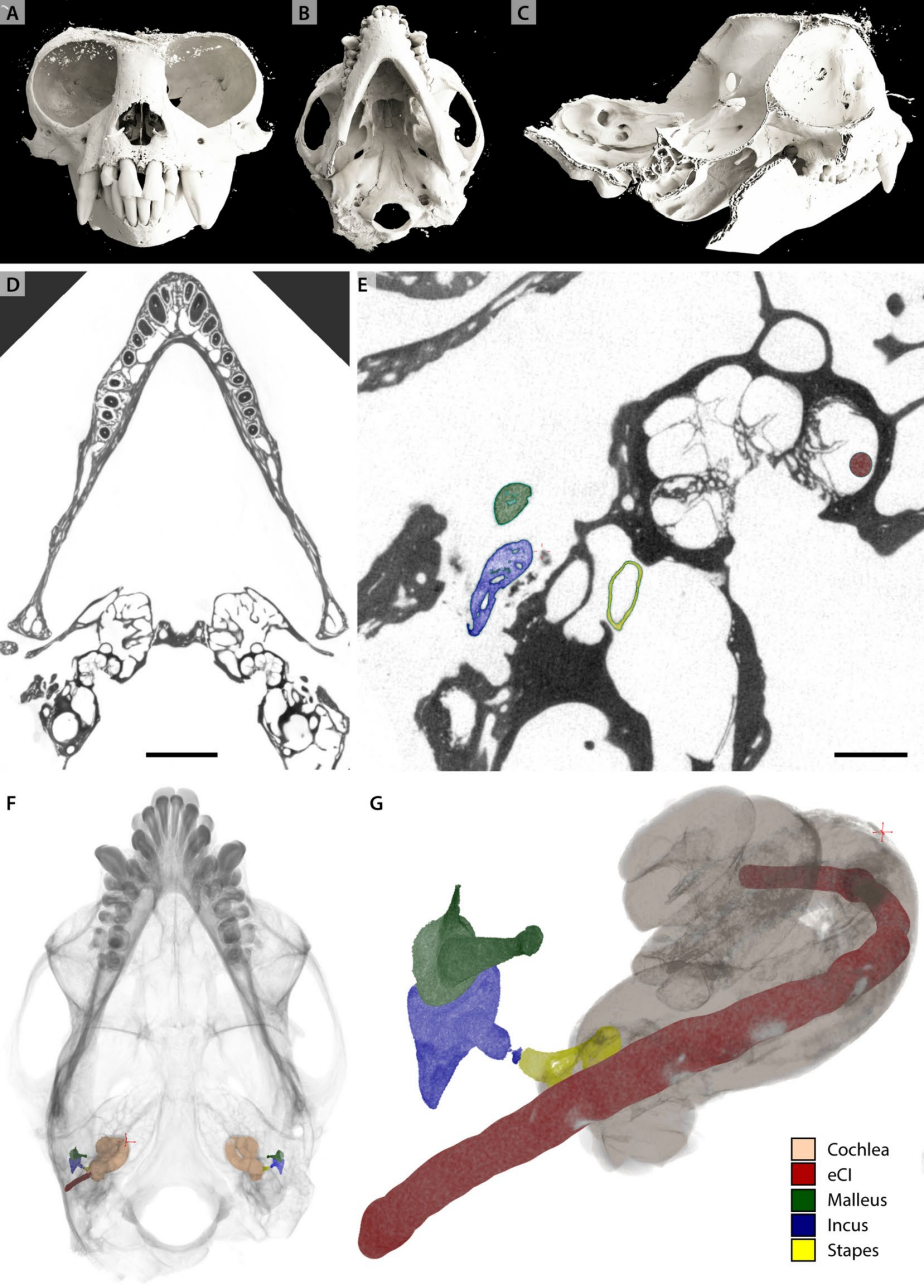
Imaging the marmoset head (sample Ma1) for eCI positioning with ${\upmu }$-CT2. (A-C) Volume rendering of the marmoset head in frontal (A), axial (B) and parasagittal (C) plane. (D) Virtual slice through the reconstruction volume of the overview scan acquired at $\textrm{dx}_\textrm{eff} = 26.4\,{\upmu }\hbox {m}$. (E) ROI-tomography at $\textrm{dx}_\textrm{eff} = 13.3\,{\upmu }\hbox {m}$, same orientation as (D). (F,G) Volume renderings of segmented cochleae, ossicles and eCI of the whole head (F) and left implanted cochlea only (G). (D,F) Scale bar 5 mm. (E,G) Scale bar 1 mm.

Finally, we present a marmoset skull (Ma1) with two intact cochleae of which the left one was implanted with an eCI during life time. The sample was scanned inhouse (${\upmu }$*-CT2*), immersed in PBS in a cylindrical jar. To accommodate high absorption by bone and implant, the sample was recorded with a high tube voltage $U_B = 120\,\hbox {kV}$. The overview scan was acquired at $\textrm{dx}_\textrm{eff} = 26.4\,{\upmu }\hbox {m}$. A subsequent ROI-tomogram was recorded at the position of the left cochlea at $\textrm{dx}_\textrm{eff} = 13.3\,{\upmu }\hbox {m}$. Fig. 9 shows slices through the virtual reconstruction volume of both scans and volume renderings of the unsegmented volume data. The bony structures and trabeculae are represented well, however soft tissue such as the OoC is not visible in this configuration. Further, both cochleae with the corresponding ossicles malleus, incus and stapes and the eCI have been segmented with region-growing and are rendered to examine eCI positioning. The eCI is placed in the basal turn of the left cochlea.

## Discussion, Summary, and Outlook

In summary, we have demonstrated multi-scale XPCT to image cochlear soft tissue. We have explored the imaging capabilities at different SR beamlines and ${\upmu }$-CT setups, selected and adjusted to provide the FOV and photon energy for various sample sizes and corresponding absorption properties. At the GINIX instrument of the coherence beamline P10 (DESY, Hamburg), we have implemented three levels of magnification with dedicated optics and detection schemes (*SR-CB2, SR-PB, SR-CB1*) for multi-scale XPCT, ranging from overview scans of the intact rodent and non-human primate cochlea, to ROI-scans at cellular resolution and even high-resolution scans of OoC tissue. At the nano-imaging beamline ID16A (*SR-CB3*) (ESRF, Grenoble), we achieved highest contrast for heavy-metal stained OoC tissue, resolving fine details of the innervating nervous tissue and fibers in 3D. While SR offers high coherence and brilliance, thus providing good contrast and high resolution with short scan times, it is limited to short beamtimes, and scheduling constraints which are not always compatible with the need to constantly and continuously support biomedical auditory research and development of implant technology. In-house ${\upmu }$-CT on the other hand can be made accessible in close proximity of pre-clinical research laboratories, in principle at all times needed within short delay. For this reason, a two-winged strategy where the SR datasets provide high quality and high contrast benchmarks, and also ground truth for a larger body of datasets acquired with ${\upmu }$-CT seems particularly well suited to address the imaging needs. Further, ${\upmu }$-CT setups offer high photon energies to cope with high absorption by mineralized bone and metal implant, for example as a validation after behavioural studies or test surgeries. While verification of the correct implant positioning can be already addressed by in-vivo scans, these can potentially also be augmented by correlation to the post-mortem higher resolution reconstructions using machine learning. Furthermore, reconstruction of bone tissue around the implant can be addressed.

Regarding sample preparation, the heavy-metal staining in combination with EPON resin embedding provides sufficient sample preservation and stability, compatible with long counting times, scanning at multiple distances for optimal phase retrieval, and relatively high dose. Note that an even more radiation resistant embedding resin has recently been reported^26^. Further, the sample is compatible with a vacuum environment. However, the photon energy has to be higher than for unstained specimen, since heavy metal stain and the EPON resin are both much more absorbing than for example the unstained OoC in methanol. To this end, we used 17.1 keV at *SR-CB3*. Note that even if the attenuation contrast was sufficient because of heavy atom labeling, one still has to apply phase retrieval when dealing with small voxel size. Otherwise, the image is blurred by near-field diffraction. When comparing reconstructions with and without phase retrieval, one clearly observes the benefit of phase retrieval even for laboratory ${\upmu }$-CT at voxel sizes in the range of a few micrometers^15^.

Liquid embedding of cochlear soft tissue immersed in methanol in small cylindrical glass tubes, on the other hand, allows imaging at lower photon energies $E = 8\,\hbox {keV}$, such as in the *SR-CB1* configuration at the GINIX endstation of P10 (DESY). However, for that dataset, a non-optimal sample preparation led to distortion of the OoC soft tissue. Also, the tubes were subject to evaporation of the alcohol and thus scanning at multiple distances and also subsequent imaging in the *SR-PB* configuration and orientation matching was not possible for all samples due to sample movement. Simple improvements of the sample environment (handling, sealing etc.) could help to solve these issues in future work. Liquid embedding of the intact cochlea in methanol or DBE already worked very well for covering the whole cochlea with ROI-tomographic scans with sub-micron voxel size in the *SR-PB* configuration. Degassing and careful mounting after transport is of importance here. For the high flux and broad spectral bandwidth at *SR-PB2*, challenges were encountered due to strong motion artefacts and bubble formation in the turns of the cochlea. This inhibited or at least compromised tomographic reconstruction for many scans. Further, long irradiation under conditions of *SR-PB2* showed structural changes and/or tomographic inconsistencies indicative of radiation damage after long acquisition. With adapted phase-retrieval schemes, imaging the optically cleared cochlea becomes also possible with ${\upmu }$-CT equipped with a liquid metal jet source (${\upmu }$*-CT1*). In short, proper choice of the proper radiation source, instrumentation, and setting of a XPCT experiment depends on the cochlea preparation and specimen size, and is not always easy. Resulting from the experience of this work, we provide a rough guideline in form of an orientation table, see Tab. 1. The indicated values, however, are only starting points and have to be carefully checked and refined in designing a study.

**Table 1:** Overview over imaging modalities and parameters recommended for different cochlea imaging applications. The values are rough estimates, intended as a guideline for selection of instrumentation and imaging protocol. Optimal and/or achievable values may change depending on the precise settings (instrumentation, geometry) and sample preparation.

application SR	FOV	modality	voxel sizes	E	scan times
unstained tissue in ethanol/PBS $^1$	1-5 mm	SR-PB	0.5-2.5 ${\upmu }$ m	8-15 keV	1 min
stained tissue in ethanol/PBS $^2$	1-3 mm	SR-PB	0.5-2.5 ${\upmu }$ m	15-30 keV	1 min
unstained small animal cochlea	1.5 mm $\times$ 5 $^1$	SR-PB stitch	0.5 ${\upmu }$ m	15-20 keV	2 h
organ of Corti, stained & disect. in raisin	<1 mm	SR-CB3	<0.5 ${\upmu }$ m	15-20 keV	30 min$^2$
organ of Corti, disect. in ethanol	<1mm	SR-CB1	<0.5 ${\upmu }$ m	8-12 keV	1 h$^2$
whole cochlea & embed.	<1 cm	SR-CB2	<10 ${\upmu }$ m	15-20 keV	10 min
application lab. $\mu$CT	FOV	modality	voxel sizes	E	scan times
whole cochlea, dried$^3$	<1 cm	${\upmu }$-CT1	<5 ${\upmu }$ m	white beam	8 h
whole cochlea, stained$^3$	<2 cm	${\upmu }$-CT2	<20 ${\upmu }$ m	white beam	4 h
small animal head & implant	<10 cm	${\upmu }$-CT2	<50 ${\upmu }$ m	white beam	2 h
$^1$: FOV enlarged by stitching $5\,\times \,5\,\times \,5$ tomograms $^2$ per defocus distance $^3$ lab. ${\upmu }$-CT requires dried or stained tissue					

In conclusion, the instrumental settings and experimental approaches described in this work may serve as a reference for future work in cochlear imaging. The overview scans as well as the stitched scans of the whole cochlea can serve as models for auditory research, in particular for tonotopic mapping. The fast imaging capabilities of the *SR-PB* setup can be exploited further for higher throughput of small-animal cochleae, thus making it possible e. g. to compare the density and number of SGNs in cochleae of different age groups and/or infer toxicology. With this study, we hope to have advanced auditory research by demonstrating the following tools/modalities and providing the corrsponding imaging workflows: (i) 3D histology with high constrast for nerve fibers and high resolution at voxel sizes down to 50 nm for stained OoC preparation (Fig.2), (ii) Whole cochlea imaging of unstained small animal cochlea with cellular and sub-cellular contrast by stitching tomograms recorded in a parallel beam, and (iii) optimized imaging instrumentation and protocols (including phase retrieval) for inhouse ${\upmu }$-CT of whole cochlea in a single acquisition. While (i) is particularly valuable for basic neuroscience, (ii) should lead us to datasets where each neuron can be localized in the entire organ, while (iii) is best suited as an imaging platform for applications such as implant development.

In future extension of this work, changes in SGN density and morphology could possibly provide insights into age-related hearing loss. Further, the high XPCT contrast even for optically cleared tissue also allows for multi-modal imaging, as already introduced in^6,7^. Finally, as the heavy-metal-stained and EPON embedded sample shows, the resolution can be increased significantly towards sub-cellular details, possibly also down to the synaptic level in future extension of this work. Multi-modal studies by X-ray imaging first and subsequent TEM (after suitable slicing or trimming) is also possible for these samples. To this end, the X-ray reconstructions could provide an overview and help to locate the regions-of-interest before studying details at highest resolution with TEM. Finally we stress, that 3D datasets presented here are available at GRO.data (DOI: 10.25625/TU53LS)^27^, for further work, e. g. in modelling.

## Methods

### Sample preparation.

All experiments complied with national animal welfare guidelines and were approved by the responsible authorities of Lower Saxony (marmoset AZ: 13/1285 and 18/2976; rodent AZ 17/2394, AZ: 20/3458). For easier orientation in this paper, the samples are referred to by identification labels (IDs) consisting of the first letters of the species and consecutive numbers, see Tab. 2 for an overview of all samples used in this manuscript.

**Table 2:** Overview of samples scanned in this XPCT study.

Parameter	Ma1	Ma2	Ma3	Ma4	Ma5	Ge1	Mo1	Mo2	WR1	WR2
Organ	head	cochlea	cochlea	cochlea	OoC tissue	cochlea	organ of Corti	organ of Corti	temporal bone	head
Decalcified	/	yes	yes	yes	yes	yes	yes	yes	/	/
Cleared	/	/	yes	yes	/	/	/	/	/	/
Implant	eCI left	/	/	/	/	/	/	/	oCI left	eCI left
Stain	/	/	/	/	/	/	heavy-metal	heavy-metal	/	/
Embedding	PBS	methanol	DBE	DBE	methanol	methanol	EPON	EPON	PBS	PBS

**Table 3:** Acquisition parameters for SR scans. $^1$: for the tissue sample Ma5 only a single tomogram was recorded.

parameter	Mo1	Mo2	Ma5	Ge1	Ma3/Ma5$^1$	Ma4	Ma4
instrument	SR-CB3		SR-CB1	SR-PB		SR-CB2	SR-PB2
(*intern*)	ID16A		P10 GINIX WG	P10 GINIX PB		P10 GINIX KB	ID17
energy *E* (keV)	17.1		8	15	13.8	13.8	50
detector	ximea		Zyla	pco		pco	pco edge 5.5
pixelsize dx (${\upmu }$m)	10		6.5	6.5		6.5	6.5
lens	10-fold		/	10-fold		2-fold	2-fold
binning	$3\times 3$		1	1		1	1
counting time (s)	0.2		2	0.035		0.5	0.035
time per tomogram (min)	53.6		65	1.2	1.2	41	2.4
# projections	2000		721 + 721	3000		1501	4001
scan range	[$0^{\circ }$, $180^{\circ }$]		[$0^{\circ }$, $180^{\circ }$], [$180^{\circ }$, $360^{\circ }$]	[$0^{\circ }$, $360^{\circ }$] (cont)		[$0^{\circ }$, $180^{\circ }$]	[$0^{\circ }$, $360^{\circ }$]
# distances	4		1	1		1	1
$z_{01}$ (m)	{16.2, 28.2, 42.1}	47.1	0.125	88		5.06	150
$z_{12}$ (m)	{1.192, 1.180, 1.166}	1.161	4.975	0.035	0.022	0.34	1
FOV per tomo ($\hbox {mm}^{2}$)	{$0.14^2,0.24^2,0.35^2$}	$0.39^2$	$0.5\times 0.35$	$1.6\times 1.4$		$8\times 7$	$9.2\times 2.3$
volume after stitching ($\hbox {mm}^{3}$)	/		/	$3.8\times 3.8\times 4.6$		/	$9.2\times 9.2\times 10.3$
voxelsize $\textrm{dx}_\textrm{eff}$ (${\upmu }$m)	{0.05, 0.08, 0.115}	0.125	0.167	0.65		3.05	3.58
# tomograms	1	1	1	$3\times 3\times 4\ (x\times y\times z)$		1	5 (*z*)
Fresnel number *F*	$\{4.2,2.4,1.4\}\times 10^{-3}$	$3.6\times 10^{-3}$	$1.8\times 10^{-3}$	0.01460	0.0207	0.3240	0.5169
imaging regime	holographic		holographic	direct contrast		direct contrast	direct contrast
phase retrieval	CTF CG		CTF	CTF		CTF	GPM
CTF $\beta /\delta$	1/27	1/15		1/45			

**Marmosets (Ma)** were bred at the DPZ (Deutsches Primatenzentrum, Göttingen, Germany). Animal **Ma1** was subjected to electrical cochlea implant (eCI) surgery. The eCI consisted of 10 electrodes with a spacing of 1 mm and was kindly provided by Med-El (Innsbruck, Austria). In a standard aseptic surgery, a retroauricular incision was performed and the muscles overlying the linea temporalis were bluntly dissected. A small mastoidectomy at and inferior to the linea temporalis was performed to expose the round window for implant insertion employing the horizontal semicircular canal and facial nerve as guiding landmarks. Next, the electrode carrier was funneled underneath the musculus temporalis from a headcap implanted in a former surgery to the mastoidectomy site. The round window niche was carefully enlarged, the round window membrane opened and the implant carefully pushed forward until all electrodes were inserted. For similar implant surgery methods, see also^28^. Afterwards the implant was fixed in place by dental acrylic at the cochlea and the electrode connector on the headcap. Several years after the implantation, the animal died suddenly for non-experimental reasons. The head was dissected and fixed in 4 % paraformaldehyde and mounted with Agarose in a cylindrical sample container.

The animals **Ma2-Ma5** (Animal IDs: Ma2 17255, Ma3 15135, Ma4 14916, Ma5 13501) were euthanized for a senescence study by veterinary pathologists. The temporal bones were dissected and fixed in 4 % paraformaldehyde (PFA) for one day. Then they were decalcified in 10 % EDTA for 13 to 18 weeks with weekly EDTA changes. The cochleae were further dissected out of the temporal bone and prepared for different imaging-techniques. **Ma2** was directly mounted in an 1.5 ml Eppendorf tube with agarose. The samples were dehydrated in a methanol dilution series to increase the concentration to 100 %.

**Ma3-Ma4** were prepared for light sheet microscopy by clearing them with an adapted form of the iDisco protocol^29^, including permeabilization, blocking with a goat serum and staining them with primary antibodies against Parvalbumin and Myosin 6. The samples were dehydrated in methanol in subsequent steps and incubated in dichloromethane to remove lipids from the sample. In the end, it was embedded in dibenzyl ether (DBE), see^7^ for further details.

**Ma5** was cut down further, exposing the turns of the cochlea and about one third of the basal turn was placed in a glass tube of diameter 1 mm and immersed in methanol.

**Mice (Mo)** were bred for research purposes at UMG and euthanized for medical training. All experiments complied with national animal care guidelines and were approved by the University of Göttingen Board for Animal Welfare and the Animal Welfare Office of the State of Lower Saxony. After removal of the cochlea, it was trimmed down to expose the OoC tissue. The tissue was fixated with 4 % PFA for 1 h. Conventional embedding of sample Mo1 was performed as previously described^30^ with 1 % OsO4 for 1 h and 1 % Uranylacetate for 1 h. Sample Mo2 was stained with 2 % OsO4 for 2 h. Subsequently, the samples were embedded in 100 % EPON. The embedding is in the form of a cylinder of diameter 1 mm.

**Gerbil (Ge)** (animal ID: 141369//85) were bred for research purposes at UMG. Gerbil **Ge1** was euthanized and the left cochlea was retrieved and immersion fixated in 4% FA. The sample was decalcified in 10% EDTA $\textrm{pH} = 8$ for several days. Subsequently, the sample was dehydrated in a methanol dilution series to increase the concentration to 100%. The samples was then mounted with agarose in an Eppendorf cup.

**Wistar rats (WR)** were bred for research purposes at UMG At the age of 6 to 7 days the animals were injected with $1\,{\upmu }\hbox {L}$ to $1.5\,{\upmu }\hbox {L}$ of AAV-PHP.B-hSyn_CatCh-EYFP_WPRE_bGH ($8.72 \times 1012\hbox {GC/mk}$) into the round window membrane. An adeno-associated virus (AAV) mediates the expression of the light-sensitive calcium translocating channelrhodopsin (CatCh)^31–33^. At the age of six months they started to undergo behavioural training. Then they were deafended using intracochlear kanamycin (100 mg/mL) injection and subjected to optical cochlea implant (oCI) surgery. During the surgery the bulla tympanica of the left ear was exposed via a retro-auricular approach and opened on an area of roughly $2\,\hbox {mm}\times 3\,\hbox {mm}$. Subsequently a cochleostomy in the basal turn of the cochlea was performed and a LED array of an oCI was inserted into the cochlea. The array was fixed at the edge of cochleostomy as well as at the bullostomy using UV glue and dental acrylic. The oCI was driven with a custom, head-mounted sound processor. After dysfunction of the oCI, the rats were euthanized under deep anesthesia via cervical dislocation. The temporal bone (**WR1**) was extracted from the skull and the LED array was cut close to the bullostomy taking care to avoid dislodging the implanted part. The sample was fixed in 4 % agarose gel and surrounded with 4 % paraformaldehyde.

Additional rats anesthesia subjected to electrical cochlea implant (eCI) surgery aiming for performance comparison between oCI- and eCI-implanted rats^33^. After dysfunction of the eCI in the behavioural study, the rats were euthanized under deep anesthesia via cervical dislocation. A whole rat head (**WR2**) including the head-worn eCI-device^34^ was fixed in 4 % agarose gel and surrounded with 4 % PFA and mounted in a cylindrical cup.

### X-ray phase-contrast tomography (XPCT)

Multi-scale XPCT was performed with a number of SR ${\upmu }$-CT instruments. SR beamline settings and parameters are tabulated in Tab. 3, and the inhouse ${\upmu }$-CT settings in Tab. 4.

**The Göttingen Instrument for Nano-Imaging with X-Rays**^9^ **(GINIX) (SR-CB1, SR-PB, SR-CB2)** is an endstation installed at the coherence beamline P10 of the PETRA III storage ring at DESY in Hamburg. X-rays are generated by a 5 m undulator located in a low beta section of the storage ring. The beam is monochromized by a double crystal Si(111) channel-cut monochromator. GINIX can be operated in different configurations.

**SR-CB1:** A X-ray waveguide (WG) with a cross section of 80 nm fabricated by e-beam lithography and wafer bonding was used as a secondary source for holographic cone beam illumination. The WG was aligned in the focus of the Kirkpatrick-Baez (KB) mirrors, at a photon energy $E = 8\,\hbox {keV}$. The waveguide exit acting as a secondary source spot provides highly coherent quasi-spherical wavefronts. Recordings at high geometric magnification are possible. Projections were recorded with an sCMOS detector ($2560\,\hbox {px}\times 2560\,\hbox {px}$, $\textrm{dx} = 6.5\,{\upmu }\hbox {m}$) with a $15\,{\upmu }\hbox {m}$ Gadox scintillator.

**SR-PB:** The GINIX instrument can also be used in a parallel beam configuration^35^, i. e. without focusing. The undulator beam passes the monochromator and directly illuminates the sample without collimating optics. Tails are cut with a sequence of slits. Single crystal attenuators were inserted to not oversaturate the detector. Images were acquired with an Optique Peter imaging system, equipped with a pco edge 5.5 camera ($2560\,\hbox {px}\times 2560\,\hbox {px}$, pixel size $\textrm{dx} = 6.5\,{\upmu }\hbox {m}$), a $50\,{\upmu }\hbox {m}$ LuAG scintillator and a 10-fold microscopic lens. Continuous rotation allowed fast scanning times, resulting in a scan times of less than 2 min per tomogram. Tomograms were acquired at a single distance, larger volumes were covered by recording multiple tomograms and stitching them together by *NRStitcher*^20^.

**SR-CB2:** Further, the instrument was operated in a non-standard manner, where the KB mirror is used to enlarge the beam to a size covering an entire marmoset cochlea, and an improvised tomographic stage was positioned directly in front of the detector about 5 m behind the KB focus, as described in^36^. Note that the low divergence of the undulator beam limits the beam size in the experimental hutch (about 87 m behind the undulator). Sample of several millimeters in size can therefore not be fully illuminated without creating a divergent beam by additional (focusing) optics. To reduce wavefront distortions by spatial filtering, a tungsten pinhole with a diameter of $3\,{\upmu }\hbox {m}$ was positioned in the KB focus. The same detector configuration as in the SR-PB setup was used, equipped with a 2-fold microscopic lens. Flatfield correction was carried with a PCA-correction algorithm^37^ to cope with intensity fluctuations.

For all three configurations, phase reconstruction was performed by the CTF approach^38^ as implemented in *HoloTomoToolbox*^39^. This was followed by a tomographic reconstruction by filtered backprojection for the parallel beam setup und FDK for the cone beam setup with the *ASTRA-Toolbox*^40,41^.

**The nano-imaging beamline ID16A (SR-CB3)**^8^ provides a beam focused by KB mirrors with high brilliance and low divergence. The energy can be set to 17.1 keV or 33.6 keV with a maximum photon (ph) flux of $4.1\times 10^{11}\,\hbox {ph/s}$. The scans were acquired at 17.1 keV at a propagation distance $z_{12} = 1.2\,\hbox {m}$ in a cone beam geometry. Projection images were recorded with a lens coupled XIMEA sCMOS detector with $6144\,\hbox {px}\times 6144\,\hbox {px}$, a physical pixelsize $\textrm{dx} = 10\,{\upmu }\hbox {m}$, equipped with a 10-fold microscopic lens and a $20\,{\upmu }\hbox {m}$ LSO scintillator. Exploiting the geometric magnification and 3-fold binning, effective pixelsizes $\textrm{dx}_{\textrm{eff}}\in \{50\,\hbox {nm}, 80\,\hbox {nm}\,\hbox {and}\,115\,\hbox {nm}\}$ were chosen. Tomographic scans with 2000 projection images were acquired at four distances to account for zeros in the contrast transfer function^24^. CTF-based phase retrieval was performed by a conjugate gradient approach^42^, followed by tomographic reconstruction using the *Nabu* software package (ESRF). The samples were scanned in a vacuum environment, sample Mo2 at room temperature and sample Mo1 in a cryogenic chamber at $-170^{\circ }\hbox {C}$.

**The ID17 beamline, ESRF (SR-PB2)** installed at a wiggler source provides a broad (white beam) spectrum^10^. Scans were acquired at a peak energy $E = 50\,\hbox {keV}$ at two resolution levels, using a microscope-coupled imaging system (Optique Peter), equipped with a sCMOS PCO edge 5.5 camera, and an interchangable objective (revolver). Low resolution scans were acquired at $\textrm{dx}_\textrm{eff} = 3.58\,{\upmu }\hbox {m}$ and high resolution scans at $\textrm{dx}_\textrm{eff} = 0.71\,{\upmu }\hbox {m}$. zSubsequent phase retrieval was performed with generalized Paganin method (GPM)^25^, followed by ring removal filtering and tomographic reconstruction (*HoloTomoToolbox*^39^, *ASTRA-Toolbox*^40,41^). The high-resolution reconstructions exhibited severe artifacts due to radiation induced bubble formation.

**The liquid-metal jet setup (**${\upmu }$**-CT1)** consists of a source (D2, Excillum, Stockholm, Sweden) with the alloy Galinstan as anode, which is liquid at room temperature. The spectrum has a strong contribution of the Ga-K$_\alpha$-line at 9.25 keV. The source was operated at 100 W electron beam power, a voltage of 70 kV, and a spot size of 10 ${\upmu }$ m x 40 ${\upmu }$ m (FWHM), resulting in a projected spot size of $10\,{\upmu }\hbox {m}\times 10\,{\upmu }\hbox {m}$. Images were acquired with a flat panel detector (Dexela 1512, Perkin Elmer, Waltham, MA, USA) with a Gadox scintillator and a physical pixelsize $dx = 75\,{\upmu }\hbox {m}$. Geometric magnification was exploited to adapt the FOV to the sample size and desired resolution. A detailed description of the custom-built instrument is given in^11,43^. Phase retrieval was performed with the *Bronnikov-aided correction (BAC)* algorithm^23^, implemented in the *HoloTomoToolbox*^39^, followed by ring removal filtering and tomographic cone-beam reconstruction with *ASTRA-Toolbox*^40,41^.

**The fixed-target anode setup (**${\upmu }$**-CT2)** is a commercial instrument (EasyTOM, RX Solutions) which can be operated with a (alternatively selectable) microfocus source (Hamamatsu L12161-07, W Target, 5-50 ${\upmu }$m spotsize) and a nanofocus source (Hamamatsu L10711-02, W target, $\textrm{LaB}_6$ cathode). Images are recorded with a flat panel detector ($1440\,\hbox {px}\times 1704\,\hbox {px}$, $\textrm{dx} = 127\,{\upmu }\hbox {m}$) or a CCD camera ($4007\,\hbox {px}\times 2672\,\hbox {px}$, $\textrm{dx} = 9\,{\upmu }\hbox {m}$) which is fibre-coupled to a Gadox scintillator. The tomographic cone beam reconstruction was performed with the software of the instrument. If needed, a filter was applied to compensate edge enhancement by phase effects.

**Table 4:** Acquisition parameters for in-house ${\upmu }$-CT scans.

parameter	Ma1 overview	Ma1 ROI	Mo2	Ma3	WR1
instrument	${\upmu }$-CT2	${\upmu }$-CT2	${\upmu }$-CT2	${\upmu }$-CT1	${\upmu }$-CT1
source	microfocus	microfocus	nanofocus	/	/
focal spot	middle	small	small	/	/
tube voltage $U_B$ (kV)	120	120	80	100	100
energy *E* (keV)	$\approx 80$	$\approx 80$	$\approx 53$	9.25	9.25
projections	1440	1440	1568	1600	1601
scan range	[$0^{\circ }$, $360^{\circ }$]	[$0^{\circ }$, $360^{\circ }$]	[$0^{\circ }$, $360^{\circ }$]	[$0^{\circ }$, $185^{\circ }$]	[$0^{\circ }$, $360^{\circ }$]
detector	flat panel	flat panel	CCD	flat panel	flat panel
pixelsize $\textrm{dx}$ (${\upmu }$m)	127	127	9	75	75
binning	/	/	2	/	/
counting time (s)	$30\times 0.4$	$7\times 4$	30	$20\times 0.8$	$10\times 0.9$
scan time (h)	13	14.5	13	24	13.5
$z_{01}$ (mm)	64.96	64.96	3.17	100	230
$z_{02}$ (mm)	312.93	632.94	128.37	1804	1054
magnification M	4.82	9.75	40.48	18.04	4.58
voxelsize $\textrm{dx}_\textrm{eff}$ (${\upmu }$m)	26.36	13.03	0.44	4.2	16.4
FOV ($\hbox {mm}^{2}$)	$31.7\times 45.4$	$17.2\times 23.6$	$0.6\times 0.9$	$6.39\times 8.08$	$25.14\times 31.81$
Fresnel number *F*	871.26	188.07	2.75	1.3651	11.1109
phase retrieval	/	/	instrument	BAC	BAC

### Data analysis.

Renderings of unsegmented data were performed with the 3D volumetric interactive visualization SDK *NVIDIA IndeX*. In addition, some unsegmented image data was also rendered with the 3D photorealistic software Siemens Cinematic Anatomy. Segmentation of SGNs and spiral ganglion neurons (SGNs) was performed with the so-called blob finder algorithm of Arivis. The blob finder algorithm is based on automated seed finding and the watershed algorithm^44^. Segmentation of the temporal bones and animal heads with a CI was performed with the software *VG Studio Max* by using the region-growing tool. All software packages that were used in the present work are tabulated in Tab. 5.

**Table 5:** List of all software packages that were used throughout this manuscript.

Matlab 2020a (2020), Mathworks, Natick, MA, USA	
https://www.mathworks.com/products/matlab.html	
HoloTomoToolbox (2020), Institute for X-Ray Physics, University of Göttingen, Germany^39^	
https://gitlab.gwdg.de/irp/holotomotoolbox	
ASTRA-Toolbox ASTRA (v. 2.1, 2022), imec-Vision Lab, University of Antwerp, Belgium^40,41^	
https://astra-toolbox.com	
Nabu tomography software (v. 2024.1.0), ESRF, Grenoble, France	
https://gitlab.esrf.fr/tomotools/nabu	
NVIDIA IndeX (2022), NVIDIA Corp., Santa Clara, CA, USA	
https://developer.nvidia.com/index	
Siemens Cinematic Anatomy (2023), Siemens Healthineers AG, Erlangen, Germany	
https://www.siemens-healthineers.com/digital-health-solutions/cinematic-rendering	
Arivis Vision 4D (v. 3.6, 2022), ZEISS Microscopy GmbH, Oberkochen, Germany	
https://www.zeiss.com/microscopy/en/products/software/arivis-pro.html	
VolumeGraphics StudioMax (v. 2023.2), Hexagon AB, Stockholm, Sweden	
https://www.volumegraphics.com/en/products/vgsm.html	
Python 3 (v. 3.9.0, 2022), Python Software Foundation, Beaverton, OR, USA	
https://www.python.org/downloads/release/python-390	
NRStitcher (non-rigid stitching), Arttu Miettinen^20^	
https://github.com/arttumiettinen/pi2	

## Supplementary Information

Supplementary Information.

## Data Availability

The reconstructed data and the analysis scripts are publically availabe at https://data.goettingen-research-online.de [https://doi.org/10.25625/TU53LS].

## Declarations

### Use of experimental animals

Research has been carried out according to the ARRIVE guidelines, where applicable.

## References

[1] Salditt, T., Aspelmeier, T. & Aeffner, S. *Biomedical Imaging: Principles of Radiography, Tomography and Medical Physics* (Berlin; Boston, De Gruyter Graduate (De Gruyter, 2017).

[2] Töpperwien, M., Markus, A., Alves, F. & Salditt, T. Contrast enhancement for visualizing neuronal cytoarchitecture by propagation-based x-ray phase-contrast tomography. *NeuroImage* **199**, 70–80. 10.1016/j.neuroimage.2019.05.043 (2019). 31129306 10.1016/j.neuroimage.2019.05.043

[3] Saccomano, M. et al. Synchrotron inline phase contrast µCT enables detailed virtual histology of embedded soft-tissue samples with and without staining. *J. Synchrotron Radiat.* **25**, 1153–1161. 10.1107/S1600577518005489 (2018). 29979177 10.1107/S1600577518005489

[4] Bartels, M., Krenkel, M., Cloetens, P., Möbius, W. & Salditt, T. Myelinated mouse nerves studied by X-ray phase contrast zoom tomography. *J. Struct. Biol.* **192**, 561–568. 10.1016/j.jsb.2015.11.001 (2015). 26546551 10.1016/j.jsb.2015.11.001

[5] Kuan, A. T. et al. Dense neuronal reconstruction through X-ray holographic nano-tomography. *Nat. Neurosci.* **23**, 1637–1643. 10.1038/s41593-020-0704-9 (2020). 32929244 10.1038/s41593-020-0704-9 PMC8354006

[6] Sagar, M. M. R. et al. Optical clearing: An alternative sample preparation method for propagation based phase contrast µCT. *Front. Phys.* **12**, 1433895. 10.3389/fphy.2024.1433895 (2024).

[7] Keppeler, D. et al. Multiscale photonic imaging of the native and implanted cochlea. *Proc. Natl. Acad. Sci. USA* **118**, e2014472118. 10.1073/pnas.2014472118 (2021). 33903231 10.1073/pnas.2014472118 PMC8106341

[8] da Silva, J. C. et al. Efficient concentration of high-energy x-rays for diffraction-limited imaging resolution. *Optica* **4**, 492–495. 10.1364/OPTICA.4.000492 (2017).

[9] Salditt, T. et al. Compound focusing mirror and X-ray waveguide optics for coherent imaging and nano-diffraction. *J. Synchrotron Radiat.* **22**, 867–878. 10.1107/S1600577515007742 (2015). 26134789 10.1107/S1600577515007742

[10] Mittone, A. et al. Multiscale pink-beam microCT imaging at the ESRF-ID17 biomedical beamline. *J. Synchrotron Radiat.* **27**, 1347–1357. 10.1107/S160057752000911X (2020). 32876610 10.1107/S160057752000911X

[11] Bartels, M., Hernandez, V. H., Krenkel, M., Moser, T. & Salditt, T. Phase contrast tomography of the mouse cochlea at microfocus x-ray sources. *Appl. Phys. Lett.* **103**, 083703. 10.1063/1.4818737 (2013).

[12] Rau, C. & Richter, C.-P. Imaging cochlear soft tissue displacement with coherent x-rays. *Phys. Scr.* **90**, 108006. 10.1088/0031-8949/90/10/108006 (2015).

[13] Glueckert, R. et al. Visualization of the membranous labyrinth and nerve fiber pathways in human and animal inner ears using MicroCT imaging. *Front. Neurosci.* **12**, 501. 10.3389/fnins.2018.00501 (2018). 30108474 10.3389/fnins.2018.00501 PMC6079228

[14] Richter, C.-P. *et al.* Evaluation of neural cochlear structures after noise trauma using x-ray tomography. In *Developments in X-Ray Tomography IX*, vol. 9212, 213–219. 10.1117/12.2062385(SPIE, 2014).

[15] Schaeper, J. J., Liberman, M. C. & Salditt, T. Imaging of excised cochleae by micro-CT: Staining, liquid embedding, and image modalities. *J. Med. Imaging* **10**, 053501. 10.1117/1.JMI.10.5.053501 (2023). 10.1117/1.JMI.10.5.053501 PMC10519431 37753271

[16] Töpperwien, M. et al. Propagation-based phase-contrast x-ray tomography of cochlea using a compact synchrotron source. *Sci. Rep.* **8**, 4922. 10.1038/s41598-018-23144-5 (2018). 29563553 10.1038/s41598-018-23144-5 PMC5862924

[17] Li, H. et al. Three-dimensional tonotopic mapping of the human cochlea based on synchrotron radiation phase-contrast imaging. *Sci. Rep.* **11**, 4437. 10.1038/s41598-021-83225-w (2021). 33627724 10.1038/s41598-021-83225-w PMC7904830

[18] Li, H. et al. synchrotron radiation-based reconstruction of the human spiral ganglion: Implications for cochlear implantation. *Ear Hear.* **41**, 173–181. 10.1097/AUD.0000000000000738 (2020). 31008733 10.1097/AUD.0000000000000738

[19] Li, H. et al. Vestibular organ and cochlear implantation: A synchrotron and micro-CT Study. *Front. Neurol.* **12**, 663722. 10.3389/fneur.2021.663722 (2021). 33897611 10.3389/fneur.2021.663722 PMC8058461

[20] Miettinen, A., Oikonomidis, I. V., Bonnin, A. & Stampanoni, M. NRStitcher: Non-rigid stitching of terapixel-scale volumetric images. *Bioinformatics* **35**, 5290–5297. 10.1093/bioinformatics/btz423 (2019). 31116382 10.1093/bioinformatics/btz423

[21] Eckermann, M. et al. Three-dimensional virtual histology of the human hippocampus based on phase-contrast computed tomography. *Proc. Natl. Acad. Sci. USA* **118**, e2113835118. 10.1073/pnas.2113835118 (2021). 34819378 10.1073/pnas.2113835118 PMC8640721

[22] Frost, J. et al. 3d virtual histology reveals pathological alterations of cerebellar granule cells in multiple sclerosis. *Neuroscience* **520**, 18–38. 10.1016/j.neuroscience.2023.04.002 (2023). 37061161 10.1016/j.neuroscience.2023.04.002

[23] De Witte, Y., Boone, M., Vlassenbroeck, J., Dierick, M. & Van Hoorebeke, L. Bronnikov-aided correction for x-ray computed tomography. *J. Opt. Soc. Am. A* **26**, 890. 10.1364/JOSAA.26.000890 (2009). 10.1364/josaa.26.000890 19340263

[24] Cloetens, P. et al. Holotomography: Quantitative phase tomography with micrometer resolution using hard synchrotron radiation x rays. *Appl. Phys. Lett.* **75**, 2912–2914. 10.1063/1.125225 (1999).

[25] Paganin, D. M. et al. Boosting spatial resolution by incorporating periodic boundary conditions into single-distance hard-x-ray phase retrieval. *J. Opt.* **22**, 115607. 10.1088/2040-8986/abbab9 (2020).

[26] Bosch, C. *et al.* 3D-Imaging of synapses in neuronal tissues with synchrotron X-ray ptychograph. 10.1101/2023.11.16.567403 (2023).

[27] Schaeper, J. J. *et al.* Replication Data for: 3D virtual histology of rodent and primate cochlea with multi-scale phase-contrast tomography. *Gro.data*. 10.25625/TU53LS (2024). 10.1038/s41598-025-89431-0 PMC11885485 40050327

[28] Dieter, A., Duque-Afonso, C. J., Rankovic, V., Jeschke, M. & Moser, T. Near physiological spectral selectivity of cochlear optogenetics. *Nat. Commun.* **10**, 1962 (2019). 31036812 10.1038/s41467-019-09980-7 PMC6488702

[29] Renier, N. et al. iDISCO: A simple, rapid method to immunolabel large tissue samples for volume imaging. *Cell* **159**, 896–910. 10.1016/j.cell.2014.10.010 (2014). 25417164 10.1016/j.cell.2014.10.010

[30] Michanski, S. et al. Mapping developmental maturation of inner hair cell ribbon synapses in the apical mouse cochlea. *Proc. Natl. Acad. Sci. USA* **116**, 6415–6424. 10.1073/pnas.1812029116 (2019). 30867284 10.1073/pnas.1812029116 PMC6442603

[31] Mager, T. et al. High frequency neural spiking and auditory signaling by ultrafast red-shifted optogenetics. *Nat. Commun.* **9**, 1750. 10.1038/s41467-018-04146-3 (2018). 29717130 10.1038/s41467-018-04146-3 PMC5931537

[32] Wrobel, C. et al. Optogenetic stimulation of cochlear neurons activates the auditory pathway and restores auditory-driven behavior in deaf adult gerbils. *Sci. Transl. Med.* **10**, eaao0540. 10.1126/scitranslmed.aao0540 (2018). 29997248 10.1126/scitranslmed.aao0540

[33] Keppeler, D. et al. Multichannel optogenetic stimulation of the auditory pathway using microfabricated LED cochlear implants in rodents. *Sci. Transl. Med.* **12**, eabb8086. 10.1126/scitranslmed.abb8086 (2020). 32718992 10.1126/scitranslmed.abb8086 PMC7611895

[34] Jablonski, L. et al. Hearing restoration by a low-weight power-efficient multichannel optogenetic cochlear implant system. *Neuroscience*[SPACE]10.1101/2020.05.25.114868 (2020).33246063

[35] Frohn, J. et al. 3D virtual histology of human pancreatic tissue by multiscale phase-contrast X-ray tomography. *J. Synchrotron Radiat.* **27**, 1707–1719. 10.1107/S1600577520011327 (2020). 33147198 10.1107/S1600577520011327 PMC7642968

[36] Reichardt, M., Frohn, J., Khan, A., Alves, F. & Salditt, T. Multi-scale X-ray phase-contrast tomography of murine heart tissue. *Biomed. Opt. Express* **11**, 2633. 10.1364/BOE.386576 (2020). 32499949 10.1364/BOE.386576 PMC7249829

[37] Hagemann, J. et al. Single-pulse phase-contrast imaging at free-electron lasers in the hard X-ray regime. *J. Synchrotron Radiat.* **28**, 52–63. 10.1107/S160057752001557X (2021). 33399552 10.1107/S160057752001557X PMC7842230

[38] Zabler, S., Cloetens, P., Guigay, J.-P., Baruchel, J. & Schlenker, M. Optimization of phase contrast imaging using hard x rays. *Rev. Sci. Instrum.* **76**, 073705. 10.1063/1.1960797 (2005).

[39] Lohse, L. M. et al. A phase-retrieval toolbox for X-ray holography and tomography. *J. Synchrotron Radiat.* **27**, 852–859. 10.1107/S1600577520002398 (2020). 32381790 10.1107/S1600577520002398 PMC7206550

[40] van Aarle, W. et al. The ASTRA Toolbox: A platform for advanced algorithm development in electron tomography. *Ultramicroscopy* **157**, 35–47. 10.1016/j.ultramic.2015.05.002 (2015). 26057688 10.1016/j.ultramic.2015.05.002

[41] van Aarle, W. et al. Fast and flexible X-ray tomography using the ASTRA toolbox. *Opt. Express* **24**, 25129. 10.1364/OE.24.025129 (2016). 27828452 10.1364/OE.24.025129

[42] Yu, B. et al. Evaluation of phase retrieval approaches in magnified X-ray phase nano computerized tomography applied to bone tissue. *Opt. Express* **26**, 11110. 10.1364/OE.26.011110 (2018). 29716036 10.1364/OE.26.011110

[43] Töpperwien, M. et al. Three-dimensional mouse brain cytoarchitecture revealed by laboratory-based x-ray phase-contrast tomography. *Sci. Rep.* **7**, 42847. 10.1038/srep42847 (2017). 28240235 10.1038/srep42847 PMC5327439

[44] McInnes, L., Healy, J. & Melville, J. UMAP: Uniform Manifold Approximation and Projection for Dimension Reduction (2020). arXiv:1802.03426.

[45] Reichmann, J. & Salditt, T. 3d structure of the human dentate gyrus by holo-tomography: Alzheimer disease vs Control. *Eur. Synchrotron Radiat. Facility*[SPACE]10.15151/ESRF-ES-527926305 (2024).

[46] Pacureanu, A. J. et al. Holographic nanotomography of metal alloys (Al-Zr-Fe-Pd) and biological samples. *Eur. Synchrotron Radiat. Facility*[SPACE]10.15151/ESRF-ES-1076345701 (2026).

