# Supplementary material for: 3D virtual histology of rodent and primate cochleae with multi-scale phase-contrast X-ray tomography
Source: Sci Rep. 2025 Mar 7;15:7933. doi: 10.1038/s41598-025-89431-0 (PMC11885485; doi:10.1038/s41598-025-89431-0)
Supplement: Supplementary file 1 — Supplementary Information. [file 41598_2025_89431_MOESM1_ESM.pdf]

Supplemental material for: 3D virtual histology of rodent and primate cochleae with multi-scale phase-contrast tomography

Jannis J. Schaeper, Christoph A. Kampshoff, Bettina J. Wolf, Lennart Roos, Susann Michanski, Torben Ruhwedel, Marina Eckermann, Alexander Meyer, Marcus Jeschke, Carolin Wichmann, Tobias Moser, Tim Salditt*

*Correspondence: tsaldit@gwdg.de

Supplemental

Additional dataset: PCA-guided flat field correction and phase retrieval in SR-CB2 Configuration

Dust or scratches on components, beam inhomogeneity or variations in the detector induce a fixed-pattern noise in the acquired projection images (p_i) that degrades the image quality and leads to challenges in the phase retrieval and artifacts in the subsequent tomographic reconstruction. For a flat field correction, additional images are acquired. Flat field images (f_i) are usually acquired before and after the tomographic scan. They are acquired with X-ray illumination, but without the sample in the beam. Dark images (d_i) are usually acquired after each scan with the X-ray source being switched off or blocked by a shutter. This signal contains the dark current of the detector. Flat fields and dark fields are acquired with the same acquisition time as the projection images. The validity and error estimation of flat field correction for the imaging regimes used are shown e. g. in[1–4].

For the conventional flat field correction, the median or mean of the flat field images $\bar{f}$ and dark images $\bar{d}$ are calculated,

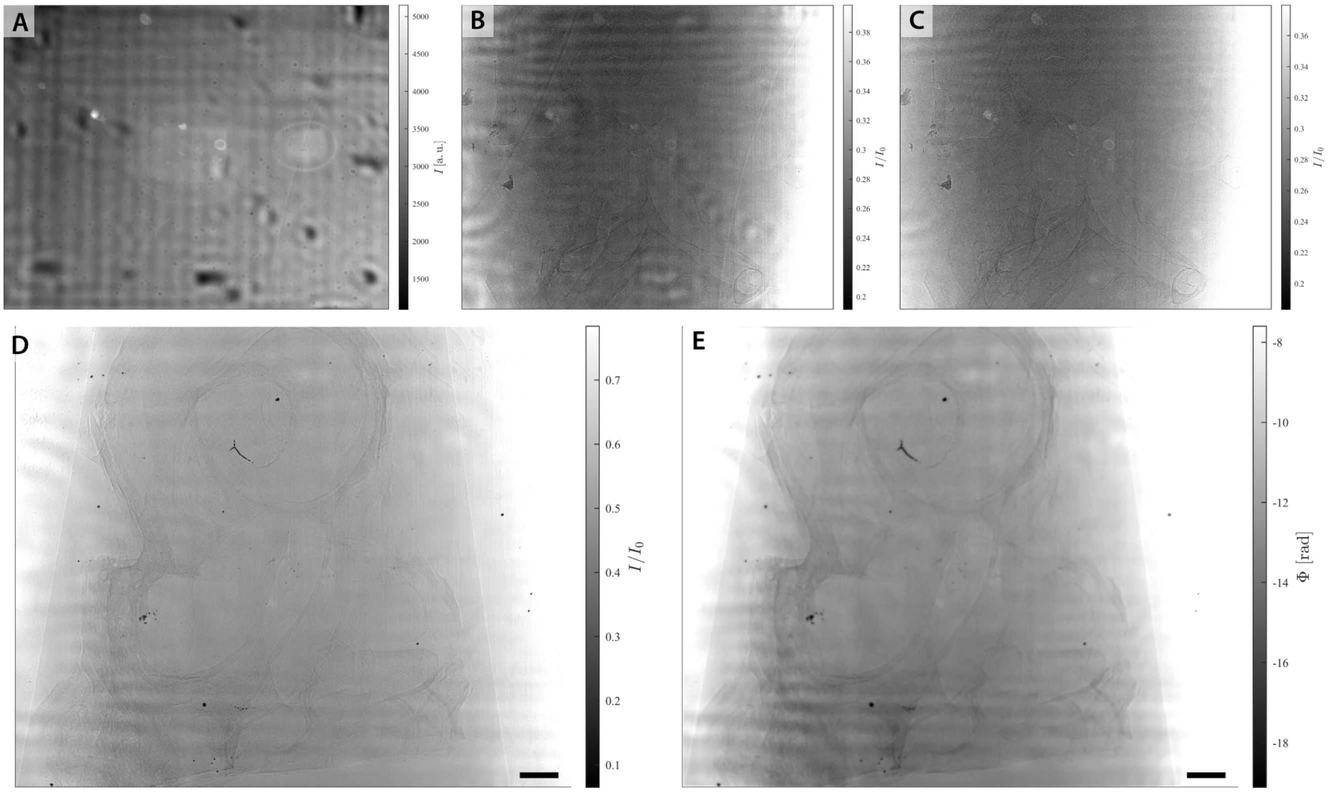

Figure 1. Flatfield correction and phase retrieval for *SR-CB2*-Setup, corresponding to the scan presented in Fig. 7(C) in manuscript. (A) Typical flatfield image. (B) XPCT projection image after division by flatfield. (C-D) XPCT projection images after PCA-aided flatfield correction. (E) Phase retrieval of (D) with CTF-scheme.

yielding the normalized (corrected) projections p':

$$p'_i = \frac{p_i - \bar{d}}{\bar{f} - \bar{d}}. \quad (1)$$

If the illumination changes linear over time, e. g. by drift of the source spot, another simple approach is to calculate a flat field for each projection by linear interpolation of the two sets of flatfield acquired before ($\bar{f}_1$) and after ($\bar{f}_2$) the acquisition of the projections

$$f_i = (1 - \frac{i}{N-1})\bar{f}_1 + \frac{i}{N-1}\bar{f}_2, \quad (2)$$

with the total number of projections N and $i \in [0, N-1]$.

For varying flat fields an efficient method is PCA-aided flat field correction where an eigen flat field is found by a linear combination of distributions obtained by principal component analysis (PCA)[5,6]:

$$f_i \approx \bar{f} + \sum_{k=0}^{K-1} w_{ik} u_k, \quad (3)$$

with K eigen flat fields u_k, $K \ll N$. Each projection is normalized by a flat field that is a linear combination of those eigen flat fields which fits the projection best. To this end, the weights w_{ik}^n are calculated by projecting the image p_i on the basis given by the eigen flat fields[5]. Fig. 1 shows the empty beam correction for a structured flatfield (A). In (B) projection image after conventional division by the mean of flatfield images is presented. With the PCA-aided approach presented in (C) the artefacts from the structured illumination are reduced.

Additional dataset: murine OoC, unstained in methanol, scanned in the SR-CB1 Configuration

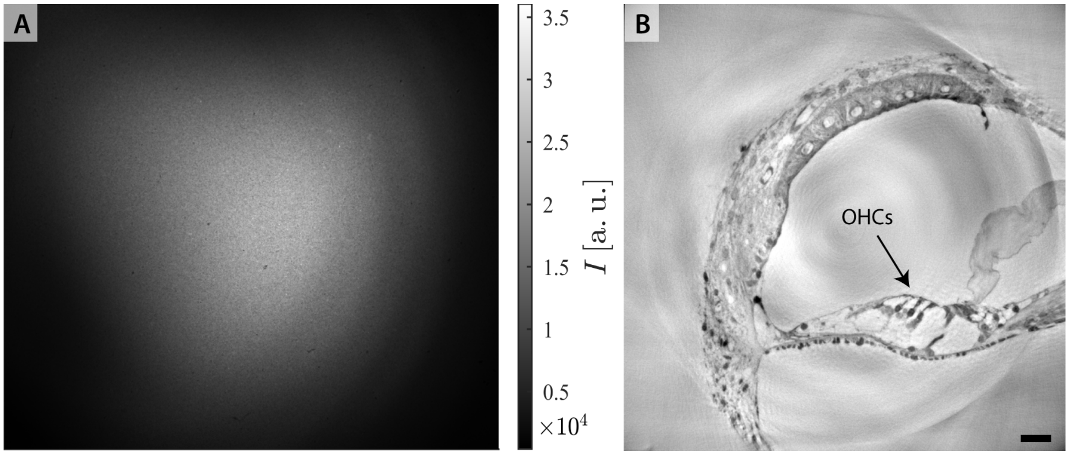

Figure 2. Flatfield correction and phase retrieval for *SR-CB2*-setup, corresponding to the scan presented in Fig. 3 in manuscript. (A) Typical flatfield image. (B) Virtual slice through reconstruction volume of mouse apical cochlear turn, recorded at $E = 8\,\text{keV}$, $\text{dx}_{\text{eff}} = 127\,\text{nm}$. Although low frequency artefacts are prevalent in the volume, single cells, such as the OHCs are resolved.

Additional dataset: custom-built instrument µ-CT1 and phase retrieval

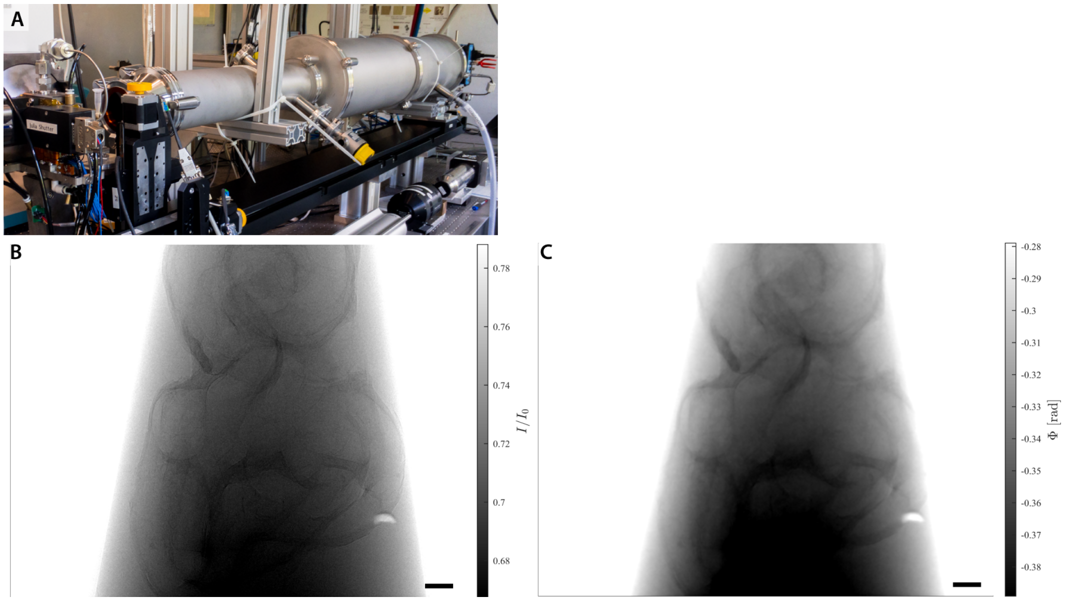

Figure 3. Setup and phase retrieval for *µ-CT1* setup, corresponding to Fig. 7(A-B) in manuscript. The sample is an optically cleared marmoset cochlea (sample Ma3) that was mounted in an Eppendorf tube with Agarose and immersed in DBE. (A) *µ-CT1*-Setup with liquid metal jet source, motorized sample stage, flighttube to reduce absorption by air and flat panel detector on detector stage. (B) Typical XPCT projection image. (C) Phase retrieval of (B) with BAC scheme.

Additional dataset: Rat Head with eCI, scanned at µCT-1

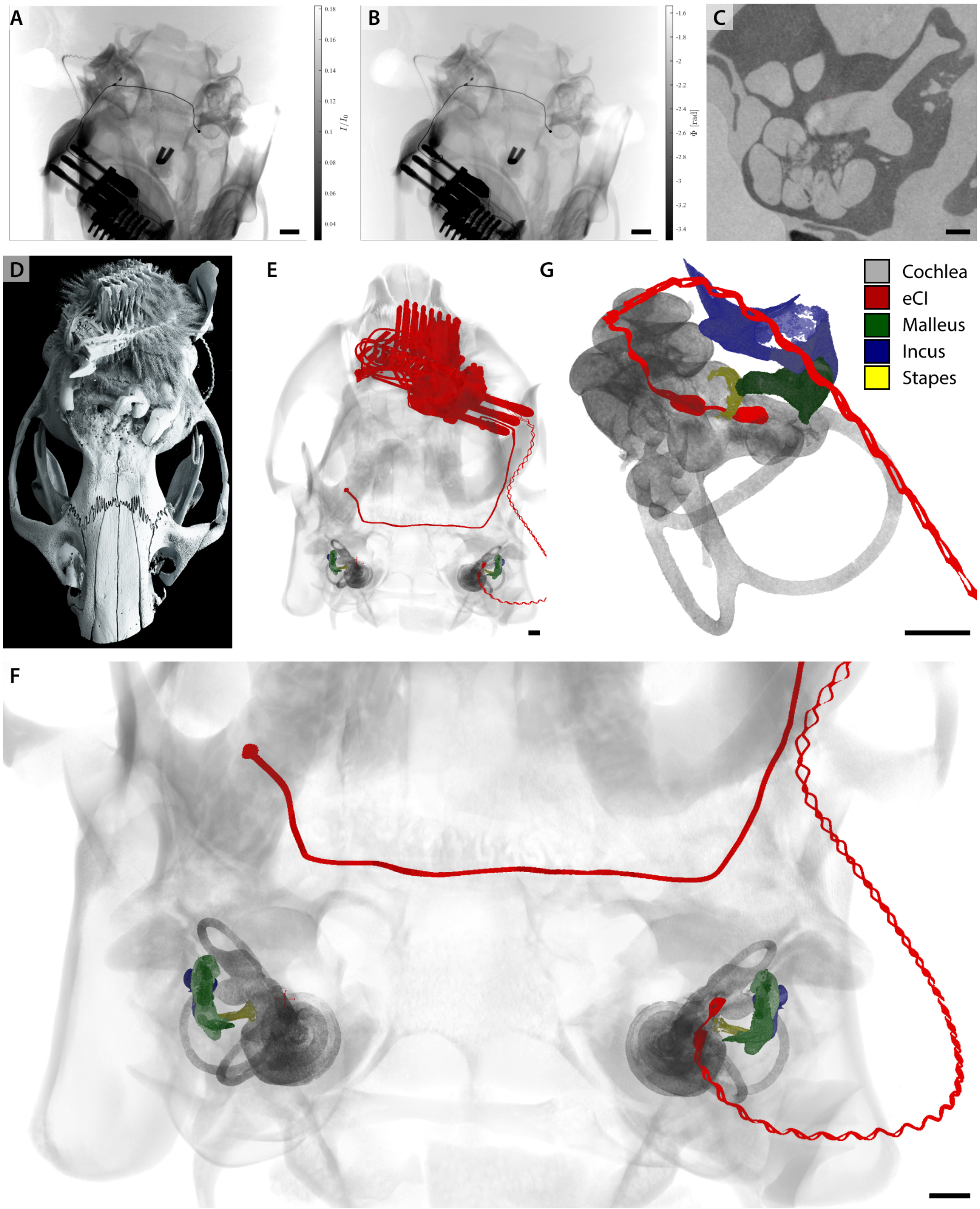

Figure 4. Imaging the rat head (sample WR2) for eCI implant positioning and defect finding with *µ-CT1* at $dx_{eff} = 16.4\,\mu m$. (A) Flatfield corrected projection. (B) Phase reconstruction of (A) with BAC scheme. (C) Virtual slice through reconstruction volume at the position of the left cochlea. (D) Volume rendering of the whole unsegmented rat head with eCI, note the metal streak artifacts at the top of the head where the implant connector sits. (E) Rendering of segmented eCI, cochleae and ossicles. (F) Zoom into (E). (G) Different orientation of the left cochlea to visualize implant positioning. (C+E+F) Share the same orientation. (A-B) Scalebars 2 mm. (C-G) Scalebars 1 mm

Additional dataset: Rat head with oCI, scanned at µCT-1

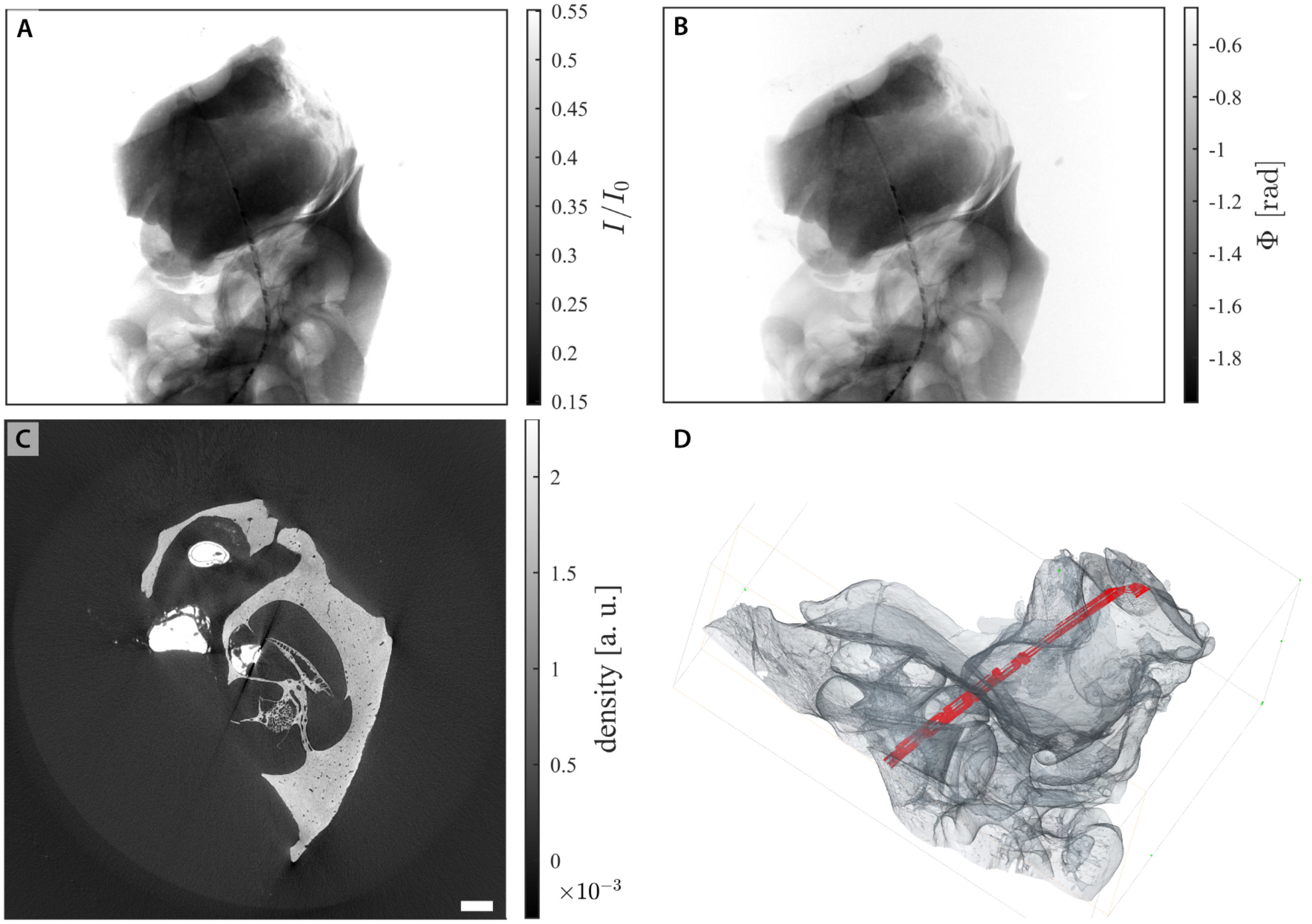

Figure 5. Imaging the rat temporal bone (sample WR1) for oCI implant positioning. (A) Typical XPCT projection image. (B) Phase retrieval of (A) with BAC scheme. (C) Virtual slice through reconstruction volume. The cochlear turns and the implant are visible. The implant causes metal streak artefacts. (D) Volume rendering of the bone (grey) and implant (red).

References

1. Homann, C., Hohage, T., Hagemann, J., Robisch, A.-L. & Salditt, T. Validity of the empty-beam correction in near-field imaging. *Phys. Rev. A* **91**, 013821, DOI: 10.1103/PhysRevA.91.013821 (2015).
2. Hagemann, J. *et al.* Reconstruction of wave front and object for inline holography from a set of detection planes. *Opt. Express* **22**, 11552–11569, DOI: 10.1364/OE.22.011552 (2014).
3. Robisch, A.-L., Wallentin, J., Pacureanu, A., Cloetens, P. & Salditt, T. Holographic imaging with a hard x-ray nanoprobe: Ptychographic versus conventional phase retrieval. *Opt. Lett.* **41**, 5519–5522, DOI: 10.1364/OL.41.005519 (2016).
4. Villanueva-Perez, P., Arcadu, F., Cloetens, P. & Stampanoni, M. Contrast-transfer-function phase retrieval based on compressed sensing. *Opt. Lett.* **42**, 1133–1136, DOI: 10.1364/OL.42.001133 (2017).
5. Hagemann, J. *et al.* Single-pulse phase-contrast imaging at free-electron lasers in the hard X-ray regime. *J. Synchrotron Radiat.* **28**, 52–63, DOI: 10.1107/S160057752001557X (2021).
6. Van Nieuwenhove, V. *et al.* Dynamic intensity normalization using eigen flat fields in X-ray imaging. *Opt. Express* **23**, 27975, DOI: 10.1364/OE.23.027975 (2015).
